# Palmitoylation-mediated regulation of KAT2A promotes lung metastasis in breast cancer

**DOI:** 10.1038/s41556-026-01913-z

**Published:** 2026-03-13

**Authors:** Ming Liu, Anke Vandekeere, Xiao-Zheng Liu, Stephan Schenck, Juan Fernández-García, Tine Tricot, Yiming Peng-Winkler, Margherita Demicco, Dorien Broekaert, Ines Vermeire, Janine Theile, Gitte Zels, Anirudh Pabba, Christine Desmedt, Janine D. Brunner, Patricia Altea-Manzano, Sarah-Maria Fendt

**Affiliations:** 1 Laboratory of Cellular Metabolism and Metabolic Regulation, VIB-KU Leuven Center for Cancer Biology, VIB, Herestraat 49, 3000 Leuven, Belgium; 2 Laboratory of Cellular Metabolism and Metabolic Regulation, Department of Oncology, KU Leuven and Leuven Cancer Institute (LKI), Herestraat 49, 3000 Leuven, Belgium; 3 VIB-VUB Center for Structural Biology, VIB, Brussels, Belgium; Structural Biology Brussels, Vrije Universiteit Brussel, VUB, Brussels, Belgium; 4 Department of Gastroenterology, Hepatology and Infectious Diseases, University Hospital Dusseldorf, Dusseldorf, Germany; 5 Laboratory for Translational Breast Cancer Research, Department of Oncology, KU Leuven, Herestraat 49, 3000 Leuven, Belgium; 6 Laboratory of Metabolic Regulation and Signaling in Cancer, Andalusian Molecular Biology and Regenerative Medicine Centre (CABIMER)- University of Seville- CSIC- University Pablo de Olavide, 41092, Seville, Spain

## Abstract

Acetylation is frequently dysregulated in cancer, and both acetyltransferase and deacetylase inhibitors are being evaluated at various stages of preclinical and clinical development. However, how the expression of acetyltransferases and deacetylases is regulated remains often elusive. We focused on the lysine acetyltransferase 2A (KAT2A) since it is important in multiple cancer indications with a clinical inhibitor in development. We discovered that *KAT2A* expression is regulated by palmitoylation in breast cancer-derived metastases. Specifically, we find that the palmitoyltransferase DHHC20 (gene name *ZDHHC20*) palmitoylates transmembrane 4 L six family member 1 (TM4SF1) promoting its plasma membrane localization. This in turn fosters phosphorylation of the signal transducer and activator of transcription 3 (STAT3), which we identify as a transcriptional regulator of *KAT2A*. Accordingly, *Zdhhc20* and *Tm4sf1* silencing as well as expression of a *Tm4sf1*double palmitoylation mutant decreases lung metastasis growth, which is rescued by *Kat2a* expression. We detect evidence of this palmitoylation-induced regulation of KAT2A in lung metastasis samples from patients with breast cancer. Thus, we show that palmitoylation can orchestrate the expression of a global acetylation regulator in lung metastases.

## Introduction

Acetylation of proteins and histones is deregulated in many cancers, which has led to multiple programs developing inhibitors against acetylating and deacetylating enzymes^1^. For deacetylating enzymes, the focus for inhibitor development has been on histone deacetylases (HDAC), with several compounds approved for the treatment of hematologic cancers^2^. Inhibition of acetylating enzymes (HATs) is less developed than for HDACs, yet clinical trials are ongoing^3^ and emerging^4^. Understanding the regulation of HDAC and HAT activity by protein interactions, auto-acetylation and phosphorylation has been important to increase the selectivity of therapeutic strategies^5, 6^. However, how the HDACs and HATs expression, which can be routinely accessed in clinical samples, is regulated in different cancers often remains poorly understood.

Palmitoylation is the attachment of palmitate to cysteine residues of proteins by a class of enzymes called palmitoyltransferases (gene name *ZDHHC*, protein name DHHC)^1^. The roles of palmitoylation and DHHC activity have been mainly studied in brain development and neurodegenerative diseases^7^. However, alterations in DHHC activity were recently also linked to cancer progression^1^. In this respect, *ZDHHC5* and *ZDHHC9* expressions have been associated with an epithelial-to-mesenchymal transition and colony-forming capacity in glioblastoma^8, 9^. In hepatocellular carcinoma, DHHC7 was reported to palmitoylate STAT3^10^. Moreover, it was shown that DHHC4 and 5 palmitoylate CD36 in breast cancer-derived lung metastases^11, 12^, while DHHC20 and 3 were found to promote pancreatic adenocarcinoma and breast cancer metastases, respectively^13, 14^. Yet, to which extent palmitoylation is intertwined with other layers of post-translational regulation is not known.

Here, we focused on the acetyltransferase KAT2A, which catalyzes histone and protein acetylation and has been identified as a promotor of cancer growth and metastasis formation in multiple cancers including breast cancer^15^, colon cancer^16^, prostate cancer^17^ and myeloid leukemia^18^. Moreover, KAT2 was identified as palmitate regulated acetyltransferase in 3D spheroids, which are an *in vitro* model for phenotypic changes required for metastasis growth^15, 19, 20^. Accordingly, KAT2 knockout has a profound effect metastasis growth, while primary tumor growth is only mildly affected in metastatic breast cancer mouse models^15^. The importance of KAT2A in tumors is further highlighted by the development of a clinical inhibitor^4^. Yet, how *KAT2A* is transcriptionally regulated is unknown.

We discovered that *KAT2A* expression is regulated by palmitoylation. Specifically, the transmembrane 4 L six family member 1 (TM4SF1) is palmitoylated by DHHC20 at two particular cysteine residues, which results in increased STAT3 phosphorylation. In turn, we identified STAT3 as a palmitate responsive regulator of *KAT2A* expression in tumor spheroids and lung metastases.

## Results

### STAT3 is a palmitate responsive transcriptional regulator of *KAT2A*

**Figure 1:**
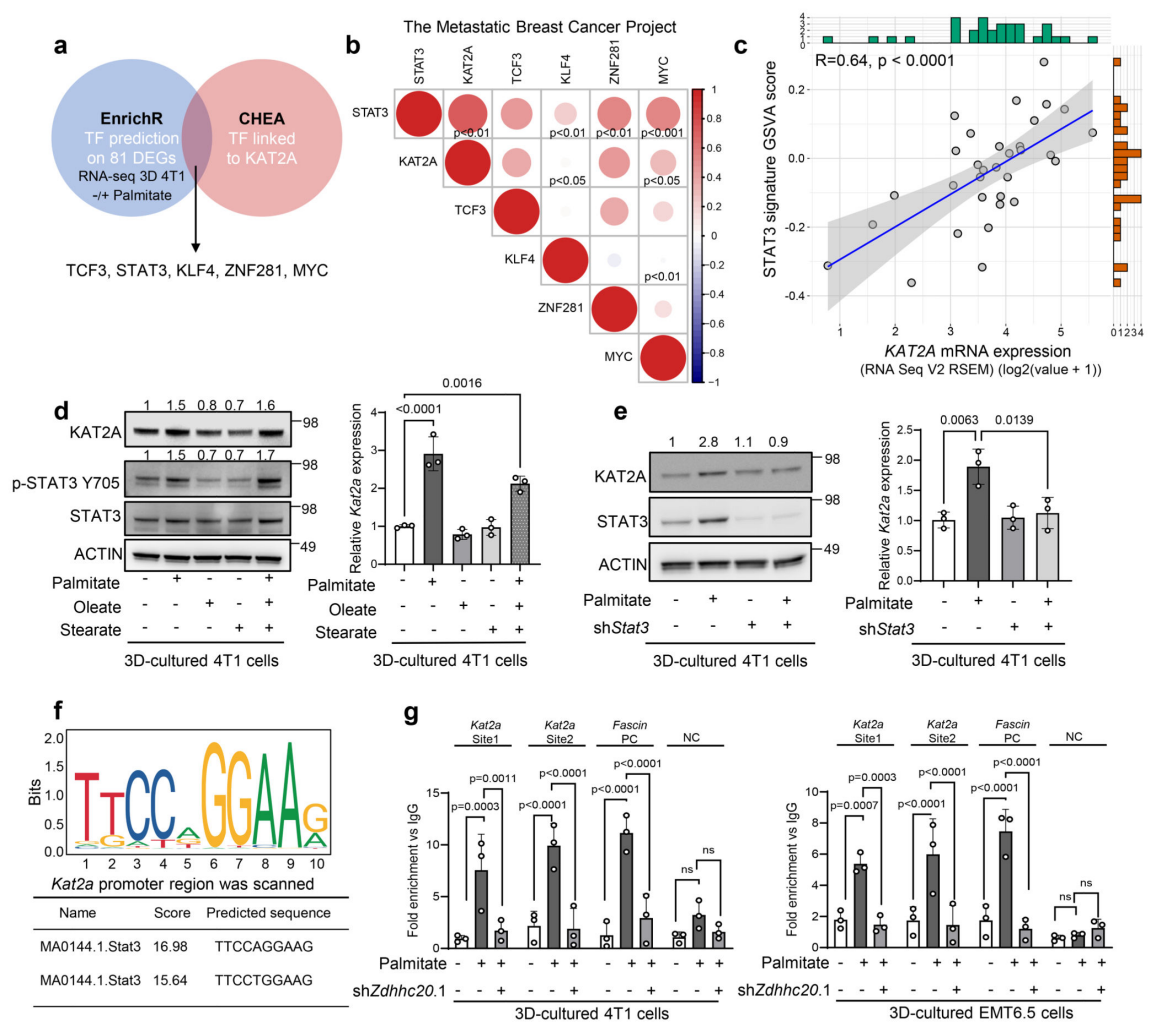
STAT3 transcriptionally regulates *KAT2A* **a**. Venn diagram of transcription factor enrichment analysis. Blue chart: TFs enrichment prediction using EnrichR based on the 81 differentially expressed genes from 3D 4T1 -/+ palmitate RNA-seq dataset. Pink chart: TFs found to be linked with *KAT2A* in the public ChIP-seq database ChEA. **b**. Pearson correlation coefficient matrix of selected transcription factors gene expression with *KAT2A* expression in breast cancer metastasis. The samples are from Metastatic Breast Cancer Project (Provisional, December 2021, n=32) RNA-seq data. Blue represents negative and red represents positive expression correlation. **c**. Pearson correlation analysis (two-tailed) between STAT3 signature GSVA score and *KAT2A* expression in the breast cancer patients metastases from cBioportal. Each dot represents one breast cancer metastasis sample; the solid line indicates the linear regression fit, and the shaded area represents the 95% confidence interval. Pearson correlation coefficient (R) and P values are shown(R = 0.645, *P* = 6.76 × 10^−5^). **d**. Protein levels of KAT2A, p-STAT3 Y705, total STAT3 and ACTIN from total lysates of 3D cultured 4T1 cells with the supplementation of palmitate (75 μM), oleate (116 μM), stearate (75 μM), and a combined condition containing all three fatty acids. The total STAT3 and ACTIN as the loading control are shown. A representative experiment is shown. n = 3 biological replicates. Numbers above the band refer to the quantification of KAT2A intensity over ACTIN; The relative mRNA level of *Kat2a* from the 4T1 cells with the supplementation of palmitate, oleate and stearate(n = 3 biological replicates). One-way ANOVA with Tukey’s multiple comparison test. Only selected statistical comparisons are shown(- vs + palmitate, *P* = 1.88 × 10^−5^). Data are presented as mean ± s.d. **e**. Protein and relative mRNA levels of KAT2A, total STAT3 and ACTIN from total lysates of 3D cultured 4T1 cells after *Stat3* knockdown with lentivirus. Numbers above the band refer to the quantification of KAT2A intensity over ACTIN. The control is 4T1 cells with non-targeting sequence pLKO vector (SCR) (n = 3 biological replicates). Two-way ANOVA with Tukey’s multiple comparison test. Only selected statistical comparisons are shown. Data are presented as mean ± s.d. **f**. Motif figure of STAT3 binding site. *Kat2a* promoter was scanned by Jaspar to predict the binding sites. Two highly scored predicted sequences were shown and used to design primers for ChIP-qPCR experiment. **g**. ChIP-qPCR assay using anti-STAT3 antibody to detect enriched gene-promoter fragments of *Kat2a* in 4T1 (left panel) and EMT6.5 (right panel) cancer cells. IgG was used as ChIP control. Values represent relative increase of real-time PCR signals compared with the signal of IgG ChIP. Two-way ANOVA with Tukey’s multiple comparison test (4T1, Kat2a site 2: Control-SCR vs Palmitate-SCR, *P* = 3.7666 × 10^−5^; Palmitate-SCR vs Palmitate-shZdhhc20.1, *P* = 2.2997 × 10^−5^; Fascin positive control: Control-SCR vs Palmitate-SCR: *P* = 9.9602×10^−7^, Palmitate-SCR vs Palmitate-shZdhhc20.1: *P* = 1.6943 × 10^−5^; Negative control: Control-SCR vs Palmitate-SCR, *P* = 0.3004; Palmitate-SCR vs Palmitate-shZdhhc20.1, *P* = 0.4907. EMT6.5, Kat2a site 2: Control-SCR vs Palmitate-SCR, *P* = 9.0440 × 10^−5^; Palmitate-SCR vs Palmitate-shZdhhc20.1, *P* = 3.9163 × 10^−5^; Fascin positive control: Control-SCR vs Palmitate-SCR: *P* = 1.2386 × 10^−6^, Palmitate-SCR vs Palmitate-shZdhhc20.1: *P* = 2.7028 × 10^−7^; Negative control: Control-SCR vs Palmitate-SCR, *P* = 0.9720; Palmitate-SCR vs Palmitate-shZdhhc20.1, *P* = 0.8501). n=3 biological replicates. Data are presented as mean ± s.d.

We asked how *Kat2a* expression is transcriptionally regulated by exploiting our previous observation that *Kat2a* expression increases in the presence of extra palmitate in tumor spheroids^15^. Specifically, we used our published RNA sequencing dataset^15^, which compared 4T1 mouse breast cancer spheroids in the presence and absence of extra palmitate (75μM). Notably, these conditions were chosen because extra palmitate increases 3D spheroid but not 2D adherent growth in 4T1 cells and cancer cell intrinsic palmitate levels and uptake are higher in 3D spheroid compared to 2D adherent cultures across multiple breast cancer cells^15^. Next, we predict transcription factors differentially active upon palmitate supplementation using EnrichR (https://maayanlab.cloud/Enrichr/) and overlayed these with gene-transcription factor associations from the ChEA database^21^. We found that KLF transcription factor 4 (*Klf4*), MYC proto-oncogene, BHLH transcription factor (*Myc*), signal transducer and activator of transcription 3 (*Stat3*), transcription factor 3 (*Tcf3*) and zinc finger protein 281 (*Znf281*) activity increased with extra palmitate (significance only for MYC and STAT3 activity signatures) and were associated with *Kat2a* expression in 4T1 spheroids (Figure 1a, Extended Data Figure 1a). To further narrow this list of possible transcription factors, we used publicly available RNA sequencing data from patients with metastatic breast cancer (The Metastatic Breast Cancer Project, www.mbcproject.org) and correlated the expression of the five identified transcription factors with *KAT2A* expression. We observed that *STAT3* expression had the strongest positive correlation with *KAT2A* expression in metastatic breast cancer patients (R=0.74, p <0.001, Figure 1b). Moreover, *KAT2A* expression was significantly correlated with a STAT3 activity signature based on the GSVA analysis across metastatic breast cancer patients (Figure 1c). Thus, we hypothesized that palmitate-induced *Kat2a* expression depends on STAT3 activity. Indeed, palmitate but not oleate or stearate supplementation increased STAT3 phosphorylation, which is indicative of its activity^22^, as well as KAT2A gene and protein expression in 4T1 spheroids (Figure 1d, Extended Data Figure 1b). Moreover, palmitate increased STAT3 phosphorylation and KAT2A expression even in the presence of oleate and stearate supplementation (Figure 1d). In line, *Stat3* silencing abrogated palmitate-induced KAT2A gene and protein expression (Figure 1e, Extended Data Figure 1c, Extended Data Figure 1d) and spheroid growth (Extended Data Figure 1e) in 4T1, and human MCF7, HCC70 and MCF10A H-Ras^V12^ breast cancer cells. Similarly, treatment with the STAT3 inhibitor Stattic (1 μM) abrogated KAT2A protein expression in 4T1 and MCF10A H-Ras^V12^ cells (Extended Data Figure 1f). Moreover, palmitate-independent activation of STAT3 phosphorylation by IL-6 was sufficient to increase *Kat2a* expression in 4T1 spheroids (Extended Data Figure 1g), confirming the positive regulation of *Kat2a* expression by STAT3. To define whether STAT3 directly regulates *Kat2a* expression we next performed ChIP-qPCR in 4T1 and EMT6.5 breast tumor spheroids with and without extra palmitate (Figure 1f). We found that STAT3 binding to the promoter regions of *Kat2a* were significantly enhanced in the presence of extra palmitate (Figure 1g). Thus, we concluded that STAT3 transcriptionally regulates *KAT2A* in the presence of extra palmitate.

### DHHC20 is required for palmitate-induced STAT3 activity

**Figure 2:**
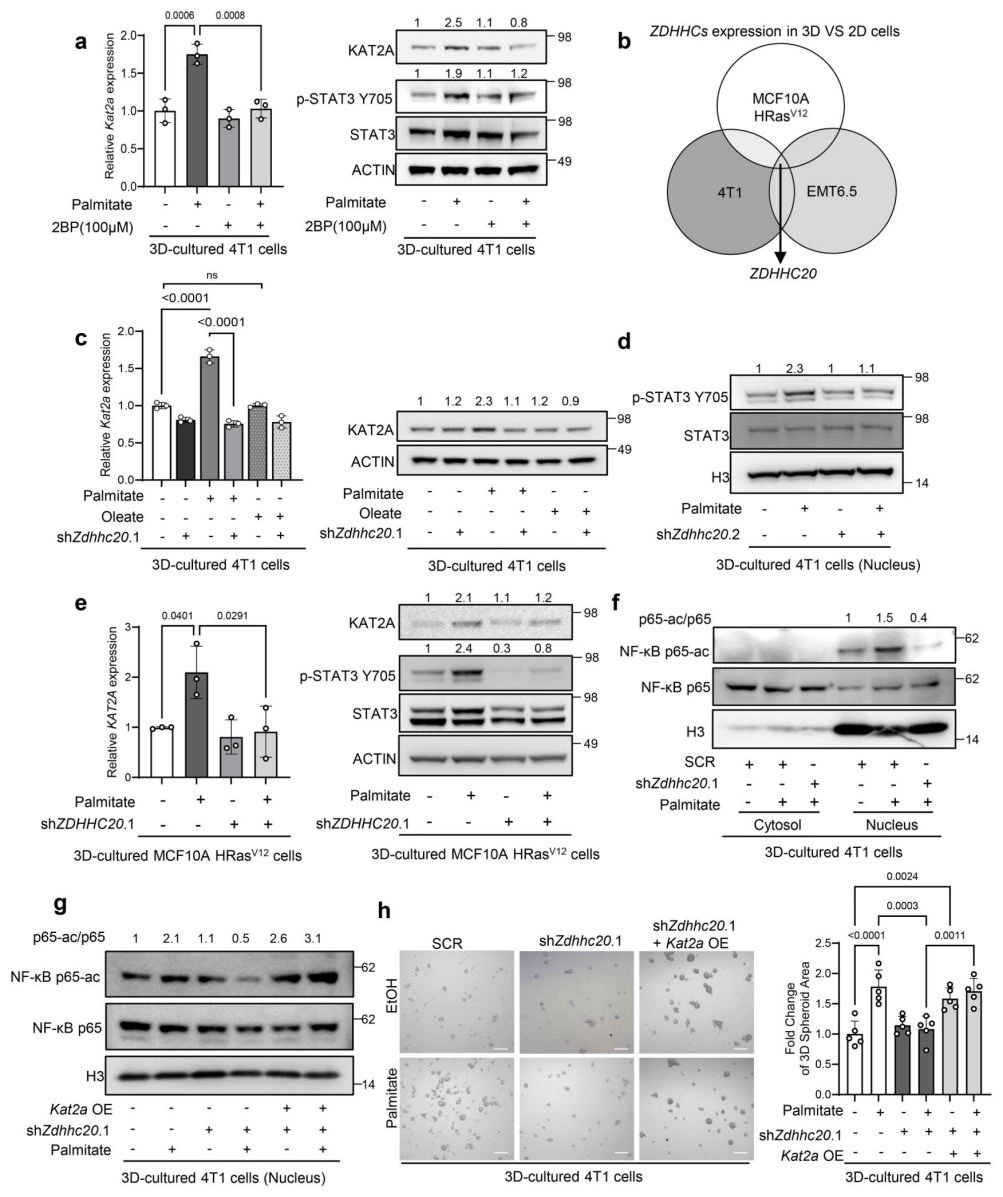
DHHC20 is required for palmitate-induced STAT3 activity **a**. Protein levels of KAT2A, p-STAT3 Y705, total STAT3 and ACTIN from total lysates of 3D cultured 4T1 cells with the supplementation of palmitate and palmitoylation inhibitor 2BP(100 μM). The total STAT3 and ACTIN as the loading control are shown. A representative experiment is shown (n = 3 biological replicates). Numbers above the band refer to the quantification of KAT2A and p-STAT3 Y705 intensity over ACTIN; The relative mRNA level of *Kat2a* from the 4T1 cells with the supplementation of palmitate and palmitoylation inhibitor 2BP (100 μM) (n = 3 biological replicates). One-way ANOVA with Tukey’s multiple comparison test. Only selected statistical comparisons are shown. Data are presented as mean ± s.d. **b**. Venn diagram of 3D vs 2D expression of ZDHHCs family in 4T1, EMT6.5 and MCF10A H-Ras^V12^ cells supplemented with palmitate (75 μM). **c**. Protein levels of KAT2A, p-STAT3 Y705, total STAT3 and ACTIN from total lysates of 3D cultured 4T1 cells with the supplementation of palmitate (75 μM) and oleate (116 μM). shRNA was used to generate *Zdhhc20* knockdown cells. The total STAT3 and ACTIN as the loading control are shown. A representative experiment is shown. n = 3 biological replicates. Numbers above the band refer to the quantification of KAT2A and p-STAT3 Y705 intensity over ACTIN; The relative mRNA level of *Kat2a* from the 4T1 cells with the supplementation of palmitate (75 μM) and oleate (116 μM) (n = 3 biological replicates). One-way ANOVA with Tukey’s multiple comparison test. Only selected statistical comparisons are shown (Control-SCR vs Palmitate-SCR: *P* = 2.87 × 10^−5^, Palmitate-SCR vs Palmitate-shZdhhc20.1: *P* = 4.54 × 10^−6^). Data are presented as mean ± s.d. **d**. Protein levels of p-STAT3 Y705, total STAT3 and Histone3 from the nucleus fraction of 3D cultured 4T1 cells. Numbers above the band refer to the quantification of p-STAT3 Y705 intensity over total Histone3. **e**. Protein levels of KAT2A, p-STAT3 Y705, total STAT3 and ACTIN from total lysates of 3D cultured MCF10A H-Ras^V12^ cells with the supplementation of palmitate (75 μM). shRNA was used to generate *Zdhhc20* knockdown cells. The total STAT3 and ACTIN as the loading control are shown. A representative experiment is shown. n = 3 biological replicates. Numbers above the band refer to the quantification of KAT2A and p-STAT3 intensity over ACTIN; The relative mRNA level of *Kat2a* from the 4T1 cells with the supplementation of palmitate. (n = 3 biological replicates). One-way ANOVA with Tukey’s multiple comparison test. Only selected statistical comparisons are shown. Data are presented as mean ± s.d. **f**. Protein levels of Ace-p65, total p65 and Histone H3 from the nucleus fraction of 3D cultured 4T1 cells with shRNA targeting *Zdhhc20*. Numbers above the band refer to the quantification of Ace-p65 intensity over total p65. **g**. Protein levels of Ace-p65, total p65 and Histone H3 from the nucleus fraction of 3D cultured 4T1 cells with shRNA targeting *Zdhhc20* and *Kat2a* overexpression as a rescue. Numbers above the band refer to the quantification of Ace-p65 intensity over total p65. **h**. 3D spheroids growth (5 d) of 4T1 cells upon *Zdhhc20* silencing using shRNA (sh*Zdhhc20*) and overexpressing *Kat2a* in the presence of extra palmitate (75 μM) represented by the average spheroids area of >100 spheroids. Data are presented as mean ± s.d. (n = 4 biological replicates). One-way ANOVA with Tukey’s multiple comparison test. Only selected statistical comparisons are shown (Control-SCR vs Palmitate-SCR: *P* = 5.99 × 10^−5^). Scale bar, 200 μm.

Next, we asked how STAT3 activity is linked to palmitate availability. One possibility is palmitoylation, which is a posttranslational modification mediated by palmitoyltransferases and palmitoyl-CoA as a substrate. In line, we found that the general palmitoylation inhibitor 2-bromopalmitate (2BP) abrogated palmitate-induced STAT3 phosphorylation and *Kat2a* expression in 4T1 spheroids (Figure 2a). Therefore, we next sought to identify the associated palmitoyltransferase. Since humans and mice have over 20 palmitoyltransferases we determined which palmitoyltransferase increases in tumor spheroid compared to 2D adherent cell cultures in mouse 4T1, because tumor spheroids are an *in vitro* model of metastasis phenotypes^15, 19, 20^ and extra palmitate increases 3D spheroid but not 2D adherent growth in 4T1 cells and cancer cell intrinsic palmitate levels and uptake are higher in 3D spheroid compared to 2D adherent cultures across multiple breast cancer cells^15^.

We observed an upregulation of *Zdhhc3, 5, 7, 15, 18, 20, 21, 24* in 4T1 spheroids compared to adherent cells based on a RNA-seq analysis (Extended Data Figure 2a). DHHC7 (protein name of *ZDHHC7*) is a known regulator of STAT3 phosphorylation in T helper 17 cells and in hepatocellular carcinoma cells^10, 23^. Therefore, we silenced *Zdhhc7* and assessed spheroid growth, STAT3 phosphorylation and KAT2A expression in the presence and absence of palmitate. Yet, we found that *Zdhhc7* silencing was not sufficient to abrogate palmitate-induced spheroid growth (Extended Data Figure 2b). In line, palmitate still increased STAT3 phosphorylation and KAT2A expression upon *Zdhhc7* silencing (Extended Data Figure 2c). Thus, we next investigated other upregulated *Zdhhc genes*. To narrow the possibilities, we examined the expression of the eight upregulated palmitoyltransferases in additional mouse EMT6.5 and human MCF10A H-RAS^V12^ cells. Among them, the expression of the palmitoyltransferase *ZDHHC20* (protein name DHHC20) consistently increased in tumor spheroids supplemented with palmitate across all cell lines (Figure 2b,Extended Data Figure 2a, 2d). Thus, we hypothesized that DHHC20 is important for the palmitate-induced tumor spheroid growth and metastasis formation. Accordingly, silencing of *Zdhhc20* decreased palmitate-induced 4T1 and MCF10A H-RAS^V12^ spheroid growth, while spheroid growth in the presence of oleate was unaffected (Extended Data Figure 2e). Moreover, we observed that *Zdhhc20* silencing abrogated the palmitate-induced increase in STAT3 phosphorylation as well as KAT2A gene and protein expression in 4T1, EMT6.5, HCC70 and MCF10A H-RAS^V12^ spheroids (Figure 2c, 2d, 2e, Extended Data Figure 2f, 2g). Notably, shRNA resistant *Zdhhc20* overexpression rescued KAT2A expression in 4T1 and EMT6.5 cells silenced for *Zdhhc20* (Extended Data Figure 2h). Next, we asked whether DHHC20 impacts the transcription factor function of STAT3. Therefore, we performed ChIP-qPCR in 4T1 and EMT6.5 tumor spheroids with and without extra palmitate upon *Zdhhc20* silencing. We observed that *Zdhhc20* silencing abrogated the palmitate-induced, but not IL-6-induced, binding of STAT3 to the *Kat2a* promotor (Figure 1g, Extended Data Figure 2i). Accordingly, IL-6 increased STAT3 phosphorylation even upon *Zdhhc20* silencing (Extended Data Figure 2j) suggesting that palmitoylation by DHHC20 is not required for the canonical IL-6-STAT3 signaling axis. Thus, we concluded that DHHC20 is required for the palmitate-induced activity of STAT3 and consequently *KAT2A* expression.

### DHHC20 is required for KAT2A activity

We have previously shown that KAT2A can activate NF-κB signaling via p65 acetylation in tumor spheroids in the presence of extra palmitate and in lung and liver metastases^15^. Therefore, we next assessed whether DHHC20 was required for palmitate-induced NF-κB signaling via p65 acetylation. Accordingly, we found that *ZDHHC20* silencing abrogated the palmitate-induced acetylation of p65 (Figure 2f). Moreover, *Kat2a* overexpression rescued p65 acetylation and 4T1 spheroids growth in the absence of DHHC20 (Figure 2g, 2h). Thus, we concluded that DHHC20 is required for KAT2A activity.

### DHHC20 palmitoylates TM4SF1 increasing its membrane localization

**Figure 3:**
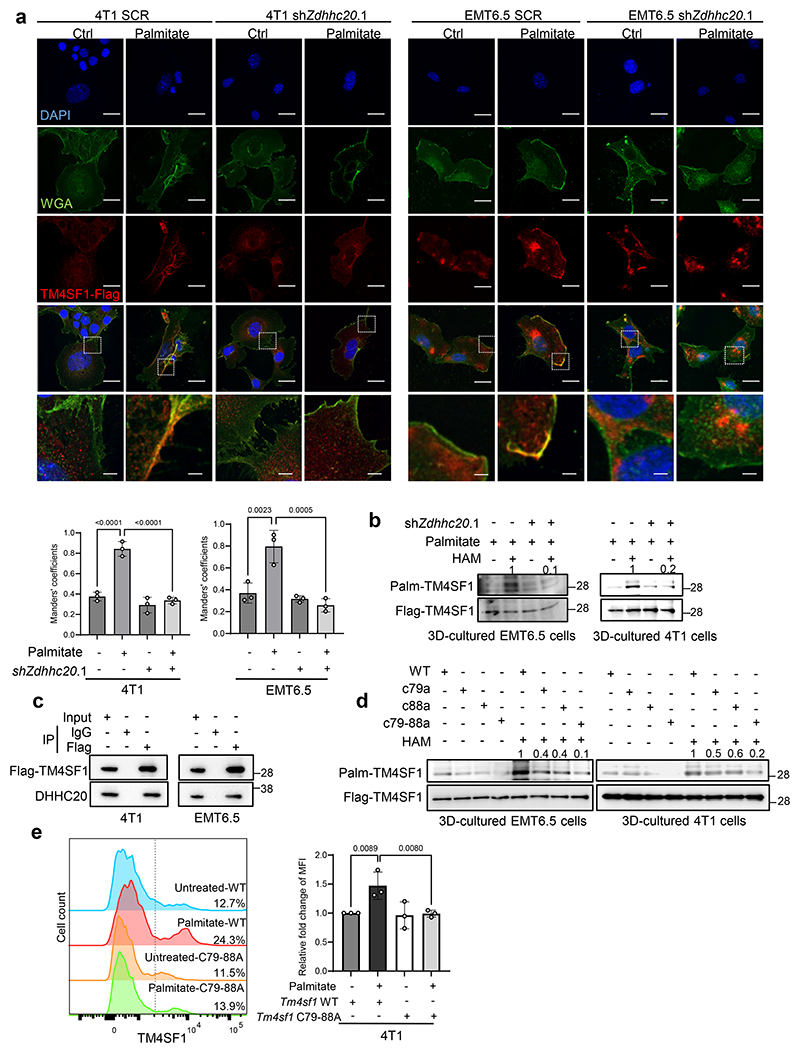
DHHC20 palmitoylates TM4SF1 increasing its membrane localization and STAT3 activation **a**. TM4SF1 localization in 4T1 and EMT6.5 cells based on immunofluorescence. Representative pictures of z-stack projections derived from 4T1 and EMT6.5 cultured in the presence or absence of palmitate(75 μM). WGA (Wheat Germ Agglutinin) was used as a membrane marker. Scale bar, 20 μm. Enlarged scale bar, 4 μm; Mander’s coefficient of Flag-TM4SF1 and WGA signals (n=3 biological replicates, mean ± s.d.), One-way ANOVA with Tukey’s multiple comparison test. Only selected statistical comparisons are shown (Control-SCR vs Palmitate-SCR: *P* = 4.76 × 10^−5^, Palmitate-SCR vs Palmitate-shZdhhc20.1: *P* = 2.63 × 10^−5^). **b**. Palmitoylation of Flag-TM4SF1 by ABE assay in the presence of PA (75 μM) with or without hydroxylamine. Numbers above the band refer to the quantification of palmitoylation intensity over total input. **c**. Co-immunoprecipitation of Flag-TM4SF1 and DHHC20 in 4T1 and EMT6.5 cells. IP, immunoprecipitation. Antibodies for IP and western blot analyses are indicated. **d**. Palmitoylation of Flag-TM4SF1 by ABE assay with the 4T1 and EMT6.5 sh*Tm4sf1* cells with the overexpression of *Tm4sf1* WT, C79A, C88A and C79-88A respectively. Numbers above the band refer to the quantification of palmitoylation intensity over total input. **e**. Flow cytometry analysis of *in vitro* cultured 4T1 cells expressing *Tm4sf1*-WT or C79-88A with or without palmitate treatment. Fold change of mean fluorescence intensity was shown to quantify the membrane expression of TM4SF1, n=3 biological replicates. Data are presented as mean ± s.d.

Next, we investigated how DHHC20 regulates STAT3 phosphorylation. It has been previously described that STAT3 can be palmitoylated^10, 23^. However, using an acyl-biotin exchange (ABE) assay which detects protein palmitoylation by exchanging the palmitoylated group to biotin labeling^24^, we could not detect a DHHC20-dependent palmitoylation of STAT3 in 4T1 tumor spheroids (Extended Data Figure 3a). Therefore, we investigated other DHHC20 targets using the overlap of two recent palmitoylation datasets^25^, which are based on a proteomics and a probe-based assay. Within the overlap of both datasets, we found transmembrane 4 L six family member 1 (TM4SF1) as a potentially palmitoylated protein that has been previously associated with STAT3 phosphorylation (Extended Data Figure 3b). Specifically, it has been shown that plasma membrane-localized TM4SF1 couples the collagen receptor tyrosine kinase DDR1 with the cortical adaptor syntenin 2, thereby connecting it to PKCα, which in turn phosphorylates and activates JAK2, initiating STAT3 activation via phosphorylation^26^ (Extended Data Figure 3c). Thus, we investigated the plasma membrane localization of TM4SF1 in breast cancer cells. Strikingly, we found that the plasma membrane localization of TM4SF1 increased in the presence of extra palmitate in 4T1 and EMT6.5 cells (Figure 3a). We next investigated TM4SF1 palmitoylation in 4T1 and EMT6.5 spheroids upon *Zdhhc20* silencing with ABE assays. We discovered that TM4SF1 is palmitoylated and found that its palmitoylation was dependent on *Zdhhc20* expression (Figure 3b). Moreover, we confirmed a direct interaction of TM4SF1 with DHHC20 in 4T1 and EMT6.5 spheroids (Figure 3c). In line, we further observed that silencing *Zdhhc20* decreased the plasma membrane localization of TM4SF1 in 4T1 and EMT6.5 cancer cells based on immunofluorescence (Figure 3a). Based on these data we concluded that DHHC20 mediated palmitoylation of TM4SF1 is required for its plasma membrane localization.

### DHHC20 palmitoylates TM4SF1 at two distinct residues

Next, we aimed to identify the DHHC20 specific palmitoylation site of TM4SF1. To do so, we utilized AlphaFold3^27^ and predicted Cys79 and Cys88 of TM4SF1 as the interaction sites with DHHC20 (Extended Data Figure 3d). Therefore, we next mutated the cysteines (C) to alanines (A) on these sites (C79A and C88A) and overexpressed single as well as double mutants in *Tm4sf1* silenced 4T1 and EMT6.5 cells. We confirmed that the double mutant protein showed very similar biochemical behavior when purified in several different detergents compared to the wild-type protein in support of correct folding, preserved oligomeric state and overall stability as well as *in vitro* DDR1 binding capacity (Extended Data Figure 3e-i), suggesting that TM4SF1 stability and function is not significantly impaired by these mutations. Consequently, we performed an ABE assay to detect palmitoylation and immunofluorescence to quantify the plasma membrane localization of TM4SF1. We observed that the single C to A mutations had only a mild impact on TM4SF1 palmitoylation and plasma membrane localization (Figure 3d, 3e, Extended Data Figure 3j). However, overexpression of the C79A+C88A double mutants displayed significantly impaired TM4SF1 palmitoylation in 4T1 and EMT6.5 tumor spheroids silenced for wildtype *Tm4sf1* (Figure 3d). Moreover, plasma membrane localization of the TM4SF1 C79A+C88A double mutants greatly decreased compared to wildtype overexpression in 4T1 and EMT6.5 tumor spheroids silenced for wildtype *Tm4sf1* (Figure 3e, Extended Data Figure 3j). Thus, we concluded that DHHC20 palmitoylates two distinct cysteines of TM4SF1 and that only the double loss of these palmitoylation sites impairs the plasma membrane localization of TM4SF1.

### TM4SF1 palmitoylation is required for STAT3 activity and KAT2A expression

**Figure 4:**
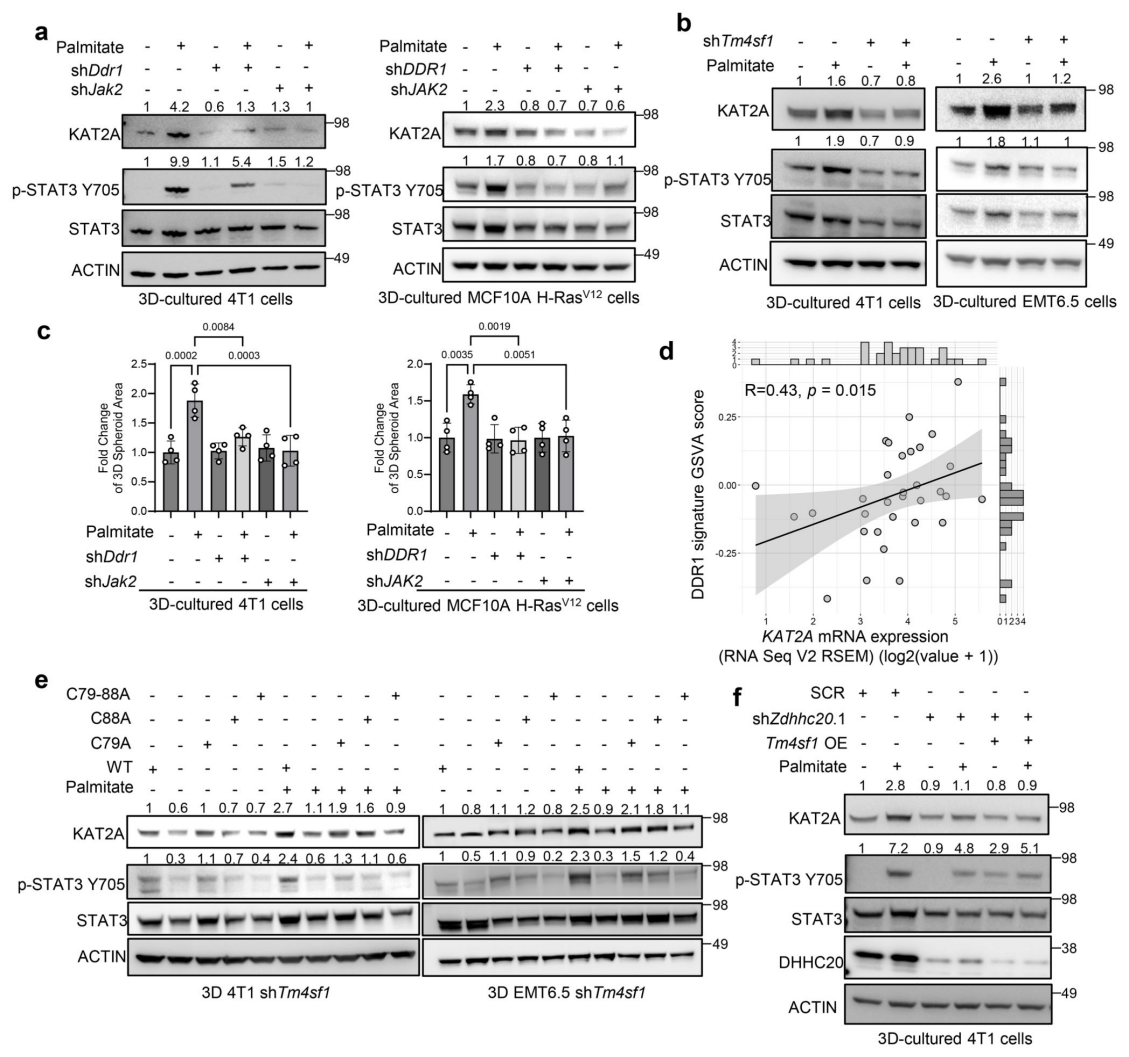
Palmitoylation of TM4SF1 requires the DDR1-TM4SF1-JAK2 axis for STAT3 activation and KAT2A upregulation. **a**. Protein levels of KAT2A, p-STAT3 Y705, total STAT3 and ACTIN from total lysates of 3D cultured 4T1 and MCF10A H-Ras^V12^ cells with the supplementation of palmitate. shRNA was used to generate *Ddr1 and Jak2* knockdown cells. The total STAT3 and ACTIN as the loading control are shown. A representative experiment is shown. n = 3 biological replicates. **b**. Protein levels of KAT2A, p-STAT3 Y705, total STAT3 and ACTIN from total lysates of 3D cultured 4T1 and EMT6.5 cells supplemented with palmitate (75 μM). shRNA was used to generate *Tm4sf1* knockdown cells. The total STAT3 and ACTIN as the loading control are shown. A representative experiment is shown. n = 3 biological replicates. **c**. 3D spheroids growth (5 d) of 4T1 cells upon *Ddr1* and *Jak2* silencing using shRNA (sh*Ddr1* and sh*Jak2*) in the presence of extra palmitate (75 μM) represented by the average spheroids area of >100 spheroids. Data are presented as mean ± s.d. (n = 4 biological replicates). One-way ANOVA with Tukey’s multiple comparison test. Only selected statistical comparisons are shown. **d**. Spearman correlation analysis (two-tailed) between DDR1 signature GSVA score and *KAT2A* expression in the breast cancer patient metastases from cBioPortal. Each dot represents one breast cancer metastasis sample; the solid line indicates the linear regression fit, and the shaded area represents the 95% confidence interval. Correlation coefficient (R) and P value are shown. DDR1 signature is based on an EO771 *Ddr1* knockdown gene set. **e**. Protein levels of KAT2A, p-STAT3 Y705, total STAT3 and ACTIN from total lysates of 3D cultured 4T1 and EMT6.5 sh*Tm4sf1* cells with the overexpression of *Tm4sf1* WT, C79A, C88A and C79-88A respectively. Numbers above the band refer to the quantification of KAT2A and p-STAT3 Y705 intensity over ACTIN. A representative experiment is shown. n = 3 biological replicates. **f**. Protein levels of KAT2A, p-STAT3 Y705, total STAT3 and ACTIN from total lysates of 3D cultured 4T1with sh*Zdhhc20* and the overexpression of *Tm4sf1* WT. Numbers above the band refer to the quantification of KAT2A and p-STAT3 Y705 intensity over ACTIN. A representative experiment is shown. n = 3 biological replicates.

Next, we asked whether TM4SF1 palmitoylation impacts palmitate-induced STAT3 phosphorylation leading to *KAT2A* expression. Activation of STAT3 signaling by TM4SF1 requires JAK2 and DDR1^26^. In line, we observed a palmitate-potentiated decrease in STAT3 phosphorylation as well as KAT2A gene and protein expression in 4T1 as well as EMT6.5 and human HCC70 as well as MCF10A H-RAS^V12^ spheroids upon *DDR1, JAK2* and/or *TM4SF1* silencing (Figure 4a, 4b, Extended Data Figure 1d). Moreover, *DDR1* and *JAK2* silencing abrogated the palmitate-induced growth in 4T1 and MCF10A H-RAS^V12^ spheroids (Figure 4c). Accordingly, JAK2 as well as PKC inhibition decreased palmitate promoted STAT3 phosphorylation and KAT2A expression (Extended Data Figure 4a), while IL-6 increased STAT3 phosphorylation even upon *Tm4sf1* silencing (Extended Data Figure 2j). Additionally, we found that a DDR1 signature GSVA score was positively correlated with *KAT2A* expression in metastases from breast cancer patients (Figure 4d). Thus, these data suggest that TM4SF1 palmitoylation may be required for the regulation of STAT3 phosphorylation and KAT2A upregulation independent of the IL-6-STAT3 axis.

To causally determine whether TM4SF1 palmitoylation is required for the palmitate-induced phosphorylation of STAT3, we overexpressed wildtype and palmitoylation mutated (C79A+C88A) *Tm4sf1* in 4T1 cancer cells silenced for wildtype *Tm4sf1*. As expected, wildtype *Tm4sf1* overexpression rescued STAT3 phosphorylation, KAT2A expression and spheroid growth (Figure 4e, Extended Data Figure 4b). However, overexpression of the *Tm4sf1* palmitoylation mutant (C79A+C88A) failed to do so in 4T1 and EMT6.5 cells silenced for *Tm4sf1* (Figure 4e, Extended Data Figure 4b). In line, overexpression of wildtype *Tm4sf1* in *Zdhhc20* silenced 4T1 tumor spheroids failed to rescue STAT3 phosphorylation, KAT2A gene and protein expression in the presence and absence of extra palmitate (Figure 4f). This shows that TM4SF1 palmitoylation is required for its plasma membrane localization and activity. Taken together, we concluded that TM4SF1 palmitoylation is required for its function as non-canonical regulator of STAT3 signaling and consequently palmitate-induced *KAT2A* expression.

### Palmitoylation is required for KAT2A promoted metastasis growth

**Figure 5:**
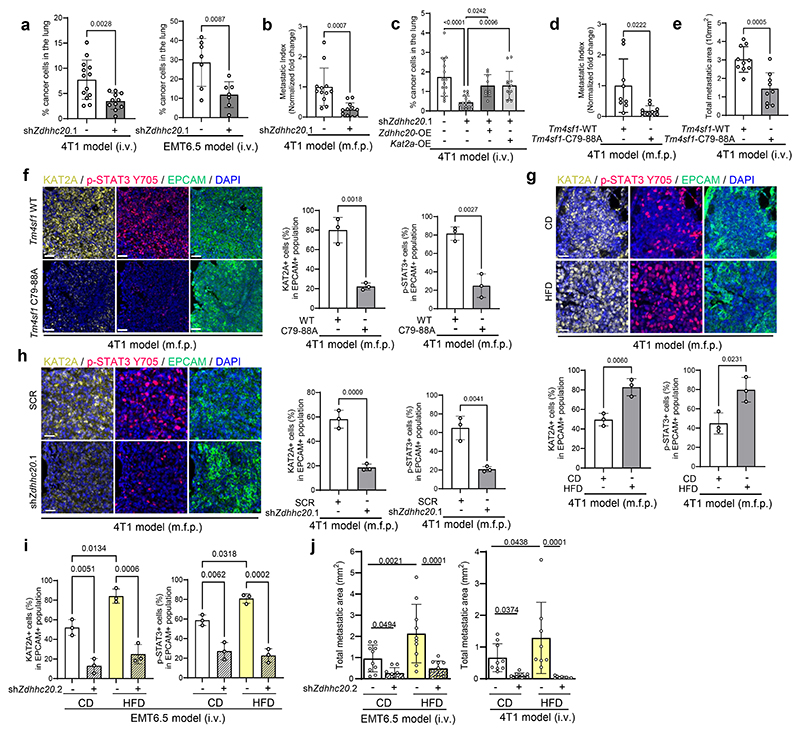
Palmitoylation promotes lung metastasis growth **a**. Left: Percentage of CD90.1^+^ 4T1 transduced with sh*Zdhhc20* (n = 11) or scramble shRNA (n = 13) in mice; Right: Percentage of CD90.1^+^ EMT6.5 cells transduced with sh*Zdhhc20* (n = 7) or scramble shRNA (n = 7) in mice. Unpaired two-tailed t-tests with Welch correction. Data are presented as mean ± s.d. **b**. Metastatic burden in the lung of mice injected with 4T1 in the m.f.p. upon genetic knockdown (n = 12 mice) of *Zdhhc20* compared to cells infected with non-targeted shRNA as a control (n = 12 mice) analyzed by H&E staining. Data are presented as mean ± s.d. Unpaired two-tailed t-tests with Welch correction. Representative H&E staining images are shown. Scale bar, 2 mm. **c**. Percentage of CD90.1^+^ 4T1 cancer cells in lung metastasis transduced with non-targeting shRNA + pLVX empty vector (n = 15 mice), sh*Zdhhc20* + pLVX-empty vector (n = 16 mice), sh*Zdhhc20* + pLVX-*Zdhhc20*-(shRNA resistant) (n = 9 mice), sh*Zdhhc20* + pLVX-*Kat2a* (n = 13 mice). One-way ANOVA with Tukey’s multiple comparison test. Only selected statistical comparisons are shown (SCR vs shZdhhc20.1: *P* = 2.58 × 10^−5^). Data are presented as mean ± s.d. **d**. Metastatic burden in the lungs of mice injected in mammary fat pad with 4T1(sh*Tm4sf1*) cells overexpressing shRNA resistant *Tm4sf1* C79a-C88A (n = 10 mice) compared to cells infected with shRNA resistant WT *Tm4sf1* as a control (n = 9 mice) analyzed by H&E staining. Data are presented as mean ± s.d. Unpaired two-tailed t-tests with Welch correction. **e**. Metastatic burden in the lung of mice intravenously injected with 4T1(sh*Tm4sf1*) overexpressing shRNA resistant *Tm4sf1* C79a-C88A (n = 9 mice) compared to cells infected with shRNA resistant WT *Tm4sf1* as a control (n = 10 mice) analyzed by H&E staining. Data are presented as mean ± s.d. Unpaired two-tailed t-tests with Welch correction. **f**. KAT2A and p-STAT3 expression in 4T1 metastases silenced for *Tm4sf1* (sh*Tm4sf1*) overexpressing wildtype (WT) *Tm4sf1* or *Tm4sf1*-C79-88A in mice (mammary fat pad injection model). Representative images of n = 3 independent mice per group. Scale bars, 50 μm**;** Percentage of KAT2A or p-STAT3 positive cells in 4T1 metastases (Number of KAT2A or p-STAT3 positive cells normalized by number of EPCAM positive cells) overexpressing WT-*Tm4sf1* or *Tm4sf1*-C79-88A. Unpaired two-tailed t-tests with Welch correction. Data are presented as mean ± s.d. **g**. KAT2A and p-STAT3 expression in 4T1 metastases in mice fed with control or high fat diet. Representative images of n = 3 independent mice per group. Scale bars, 50 μm**;** Percentage of KAT2A or p-STAT3 positive cells in 4T1 metastases (Number of KAT2A or p-STAT3 positive cells normalized by number of EPCAM positive cells). Unpaired two-tailed t-tests with Welch correction. Data are presented as mean ± s.d. **h**. KAT2A and p-STAT3 expression in 4T1 metastases with scramble or sh*Zdhhc20* transduction in mice (mammary fat pad model). Representative images of n = 3 independent mice per group. Scale bars, 50 μm**;** Percentage of KAT2A or p-STAT3 positive cells in 4T1 metastases (Number of KAT2A or p-STAT3 positive cells normalized by number of EPCAM positive cells). Unpaired two-tailed t-tests with Welch correction. Data are presented as mean ± s.d. **i**. Percentage of KAT2A or p-STAT3 positive cells normalized to EMT6.5 metastases (EPCAM positive cells) transduced with shScramble or sh*Zdhhc20* in mice fed with control (CD) or high fat diet (HFD), n = 3 biological replicates per group. Before injections, mice were fed for 16 weeks on CD and HFD. Two-way Anova with Tukey’s multiple comparison was performed. Data are presented as mean ± s.d. **j**. Metastatic burden in the lung of mice injected with 4T1 and EMT6.5 in the i.v upon genetic knockdown of *Zdhhc20* compared to cells infected with non-targeted shRNA as a control analyzed by H&E staining. Before injections, mice were fed for 16 weeks on CD and HFD (EMT6.5: SCR-CD, n = 10 mice, sh*Zdhhc20*.2-CD, n = 9 mice, SCR-HFD, n = 10 mice, sh*Zdhhc20*.2-HFD, n = 10 mice; 4T1: SCR-CD, n = 9 mice, sh*Zdhhc20*.2-CD, n = 9 mice, SCR-HFD, n = 8 mice, sh*Zdhhc20*.2-HFD, n = 9 mice). Two-way Anova with Tukey’s multiple comparison was performed. Data are presented as mean ± s.d.

Next, we investigated the importance of DHHC20 mediated palmitoylation of TM4SF1 in metastasis growth. Specifically, we injected *Zdhhc20* silenced, *Tm4sf1* palmitoylation mutant (C79A+C88A) expressing and control EMT6.5 and/or 4T1 cancer cells into the mammary fat pad and/or intravenously to mice. In line with a low expression of DHHC20 in primary tumors compared to lung metastases of mice and patients with breast cancer (Extended Data Figure 5a, 5b), we found that *Zdhhc20* silencing did not change primary tumor growth in mice (Extended Data Figure 5c, 5d). However, we observed that *Zdhhc20* silencing in cancer cells decreased lung metastasis induced by intravenous cancer cell injection (Figure 5a) as well as spontaneous metastasis from mammary fat pad injections (Figure 5b). Additionally, overexpression of *Kat2a* or *Zdhhc20* in *Zdhhc20* silenced 4T1 cells rescued metastasis growth (Figure 5c). Moreover, mutating the C79A+C88A palmitoylation sites of TM4SF1 in 4T1 cancer cells decreased metastatic burden of mice compared to control cells expressing wildtype TM4SF1 regardless of whether the cells were injected intravenously or in the mammary fat pad (Figure 5d, 5e, Extended Data Figure 5e, 5f). In line, metastases from TM4SF1 palmitoylation mutant (C79A+C88A) 4T1 cells displayed reduced KAT2A expression and STAT3 phosphorylation compared to control metastases regardless of whether the cells were injected intravenously or in the mammary fat pad (Figure 5f, Extended Data Figure 5h). Thus, we concluded that DHHC20 activity and TM4SF1 palmitoylation are required for effective lung metastasis growth.

Next, we investigated the importance of palmitoylation in high fat diet (HFD)-promoted metastasis growth. To do so, we fed mice for 4 months a HFD (20% carbohydrate, 20% protein, 60% fat). Subsequently, we injected control and *Zdhhc20* silenced 4T1 and EMT6.5 cancer cells intravenously or into the mammary fat pad of HFD or control diet fed mice. We found that the metastases of HFD fed mice showed increased STAT3 phosphorylation and KAT2A expression compared to the metastases of control diet fed mice regardless of whether the cells were injected intravenously or in the mammary fat pad (Figure 5g, Extended Data Figure 5i, 5j). Notably, knockdown of *Zdhhc20* decreased STAT3 phosphorylation and KAT2A expression in the lung metastasis (Figure 5h, 5i, Extended Data Figure 5i). Moreover, the knockdown of *Zdhhc20* in cancer cells decreased lung metastasis growth based on number, average lung metastases size and normalized area of total lung metastasis (metastasis area over total lung area) in control diet and in particular in HFD fed mice by 5 and 17-fold, respectively (Figure 5j, Extended Data Figure 5k). Thus, we concluded that targeting DHHC20-mediated palmitoylation is sufficient to decrease palmitate-induced metastasis growth.

### Evidence for palmitoylation regulation of KAT2A in breast cancer patients

**Figure 6:**
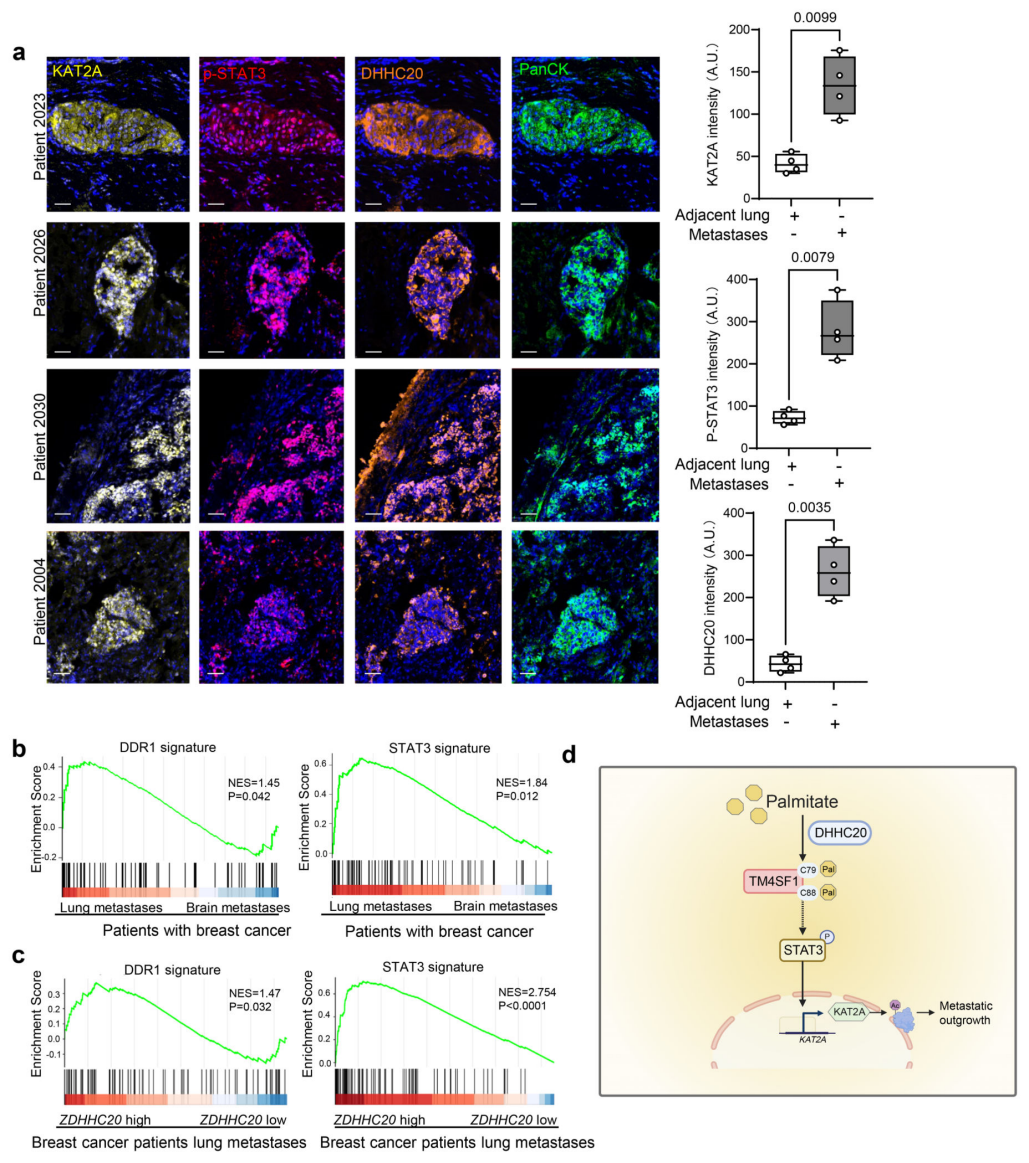
Evidence for the palmitoylation-KAT2A axis in lung metastasis from patients with breast cancer **a**. KAT2A, p-STAT3 and DHHC20 protein expression in lung metastases of breast cancer patients from the UPTIDER program by multiplex opal staining. Scale bars, 50 μm. Quantification is based on n = 4 patient samples. Box plots show the median (centre line), 25th and 75th percentiles (box), and whiskers representing the minimum and maximum values. Each dot represents an individual sample. Unpaired two-tailed t-tests with Welch correction. Exact P values are shown. **b**. GSEA enrichment plots of DDR1 and STAT3 signature in breast cancer patients’ metastases comparing lung and brain (GSE14018). NES, normalized enrichment score. P value indicates the significance of the enrichment score based on a two-sided permutation test. **c**. GSEA enrichment plots of DDR1 and STAT3 signature in breast cancer patients’ lung metastases comparing high and low *ZDHHC20* expression (GSE193103). NES, normalized enrichment score. P value indicates the significance of the enrichment score based on a two-sided permutation test (*P* = 5.44 ×10^-8^). **d**. Graphical summary of the mechanism. DHHC20 responds to extra palmitate by palmitoylating TM4SF1 increasing its membrane association, which results in the phosphorylation of STAT3 and consequently *KAT2A* expression with subsequent protein acetylation promoting metastasis. Figure was created with a licensed version of BioRender.com.

Finally, we determined key components of the described mechanism in samples from patients with breast cancer. Therefore, we collected lung metastasis tissues from four patients with breast cancer within the UPTIDER rapid autopsy program (Supplementary Table 1)^28^ and determined DHHC20 protein expression, STAT3 phosphorylation and KAT2A protein expression. We observed an increased expression of all proteins in lung metastases compared to the adjacent non-cancerous tissues (Figure 6a). Next, we analyzed palmitate-induced signaling in publicly available data from lung and brain metastases of patients with breast cancer because it was previously shown that the brain environment is lipid scarce compared to the lung environment^29–31^. Indeed, we found that signatures indicative of DDR1 and STAT3 signaling were increased in lung compared to brain metastases of breast cancer patients (Figure 6b). Moreover, when we stratified lung metastasis samples from breast cancer patients^32^ for high *versus* low *ZDHHC20* expression, we found that the gene signatures indicative of DDR1 and STAT3 signaling were enriched in lung metastasis samples with high compared to low *ZDHHC20* expression (Figure 6c). Therefore, we concluded that this provides evidence for the palmitoylation-induced KAT2A activity via STAT3 in breast cancer patients with lung metastases.

In conclusion, we discovered that palmitoylation of TM4SF1 by DHHC20 promotes STAT3 signaling and in turn transcription of the protein and histone acetyltransferase *KAT2A*. This identifies a convergence of palmitoylation and acetylation in metastasis growth.

## Discussion

Deregulated acetylation has become an attractive therapeutic strategy in cancer that is exploited in pre-clinical and clinical studies^1^. Here, we discovered that the acetyltransferase *KAT2A* is regulated by palmitoylation (Figure 6d). This interconnection between palmitoylation and acetylation shows that the availability of the fatty acid palmitate, which is influence by diet and primary tumors in patients^15, 33, 34^, not only provides acetyl-CoA, the metabolite substrate for acetylation, but can regulate the expression of the respective acetyltransferase.

Moreover, we complement the current knowledge with several aspects. While the importance of TM4SF1 mediated signaling and protein interactions in metastasis formation have been previously identified^26, 35–37^ suggesting TM4SF1 as a drug target^38–40^, we add to the current knowledge by showing that TM4SF1 needs to be palmitoylated to have sufficient plasma membrane localization and thus functionality in breast cancer metastasis growth. This suggests that TM4SF1 may be particularly important in high palmitate environments.

DHHC20 has been previously studied in metastatic pancreatic adenocarcinoma, and it was found that *Zdhhc20* knockout decreased metastasis in immunocompetent but not natural killer cell-depleted mice^16^. In addition, we discovered a cancer cell intrinsic role of DHHC20 enabling KAT2A-mediated acetylation, which could be further explored given that it was previously shown that whole body *Zdhhc20* knockout mice are viable and have no adverse phenotype or histological abnormalities as adults^16^. Substrates of DHHC20 are the epidermal growth factor receptor (EGFR) in breast cancer cells^41, 42^, which causes increased sensitivity to EGFR inhibitors as well as YTH N6-Methyladenosine RNA Binding Protein F3 (YTHDF3) in pancreatic ductal adenocarcinoma in the context of KRAS signaling^43^. Moreover, STAT3 was shown to be palmitoylated by DHHC7 in T helper 17 cells and in hepatocellular carcinoma cells^10, 23^. We in addition demonstrate that STAT3 signaling becomes palmitate responsive through TM4SF1 palmitoylation, and that this mechanism is independent of canonical STAT3 phosphorylation by IL-6. Accordingly, STAT3 exerted control over KAT2A expression only when its phosphorylation was increased in the presence of extra palmitate but not at basal phosphorylation levels without extra palmitate, which might be explained by various known regulatory mechanisms^44–48^. Thus, our finding may provide a valuable selection criteria for patient inclusion based on circulating palmitate levels in ongoing and future clinical trials of STAT3 inhibitors^49^.

KAT2A is a major regulator of protein and histone acetylation in cancers highlighting as a central hub of tumor development and progression^50^. Moreover, KAT2A has previously been suggested to act upstream as a regulator of STAT3 signaling^51, 52^. Here, we identify STAT3 as a transcriptional regulator of KAT2A, revealing a reciprocal regulatory relationship that may inform improved patient selection criteria and more selective therapeutic strategies.

In conclusion, we functionally connect palmitoylation to the regulation of protein acetylation in cancer metastasis.

## Methods

All animal experiments were conducted in compliance with the guidelines approved by the Ethical Committee of KU Leuven (Project #P059/2023). All antibodies were commercially available polyclonal antibodies validated by the manufacturers. All oligonucleotide sequences are provided and were obtained from Integrated DNA Technologies (IDT).

### Experimental Model and Study Participant Details

#### Cell Lines and Culture Conditions

MCF10A (ER-/PR-/HER2-), MCF7 (ER+/PR+/HER2-), and 4T1 (ER-/PR-/HER2-) cell lines were obtained from ATCC. The EMT6.5 (ER-/PR-/HER2-) cell line was generously provided by R. Anderson (Peter MacCallum Cancer Center). MCF10A cells expressing the oncogenic driver H-Ras^V12^ (MCF10A H-Ras^V12^) were generated as previously described^20^ to serve as a relevant *in vitro* breast cancer model, given that approximately 50% of human breast cancers exhibit increased H-Ras activity^53^. The utility of this cell line in studying metastasis biology has been previously established^20^. 4T1 and EMT6.5 cells were cultured in Roswell Park Memorial Institute (RPMI) 1640 medium supplemented with 10% fetal bovine serum (FBS), 1% penicillin (50 units/mL), and 1% streptomycin (50 μg/mL). MCF7 cells were maintained in Dulbecco’s Modified Eagle’s Medium (DMEM) supplemented with 10% FBS, 1% penicillin (50 units/mL), and 1% streptomycin (50 μg/mL). MCF10A H-Ras^V12^ cells were cultured in DMEM/F12 medium supplemented with 5% horse serum, 1% penicillin (50 units/mL), 1% streptomycin (50 μg/mL), 0.5 μg/mL hydrocortisone, 100 ng/mL cholera toxin, 10 μg/mL insulin, and 20 ng/mL recombinant human EGF. All cells were maintained at 37°C in a humidified incubator with 5% CO_2_ and 95% relative humidity. Cells were regularly tested for mycoplasma contamination using the MycoAlert Detection Kit (Lonza), with negative results.

For 3D culture conditions, soft agar-coated plates were prepared as previously described^20^. 1% soft agar was mixed 1:1 with the respective culture medium and allowed to solidify at room temperature. Cells were plated on top of the base agar and incubated for 3–5 days. We supplemented all fatty acids as conjugates with BSA. The concentration was adapted per fatty acid so that the final concentration for each fatty acid in media containing FBS added up to 130 μM. Notably different supplementations were needed because FBS contains different basal amounts of the supplemented fatty acids. A final concentration of 130 μM (corresponding to an addition of 75 μM) was chosen because for palmitate this robustly increased tumor spheroid growth^15^. The following compounds were used: Recombinant IL-6 (Proteintech, HZ-1019; R&D Systems, 406-ML), Fedratinib (MedChemExpress, HY-10409), Go 6983 (MedChemExpress, HY-13689), STATTIC (MedChemExpress, HY-13818), fatty acids: sodium palmitate, stearic acid, oleic acid (Sigma-Aldrich).

#### Generation of Genetically Modified Cell Lines

Lentiviral particles were produced in Lenti-X 293T cells via transfection with psPAX2, PMD2.G, and lentiviral vectors using Lipofectamine 2000. For shRNA-mediated knockdown, 4T1 or EMT6.5 cells were infected with lentivirus carrying pLKO.1 vectors expressing shRNAs targeting *Zdhhc20, Stat3, Tm4sf1, Ddr1, Jak2* (Belgian Coordinated Collections of Microorganisms, BCCM) and non-targeting control. For MCF10A HRAS^V12^, MCF7, and HCC70 human cell lines, shRNAs targeting *ZDHHC20, STAT3, TM4SF1, DDR1*, and *JAK2*(Belgian Coordinated Collections of Microorganisms, BCCM), as well as a non-targeting control, were used. After 24 hours, the medium was replaced, and cells were selected with puromycin(2 μg/ml for 4T1 cells, 1 μg/ml for EMT6.5 cells and 0.3 µg/ml for MCF10A HRAS^V12^) for 72 hours. For overexpression of *Kat2a, Zdhhc20 and Tm4sf1*(WT + Mutant), the pLVX-IRES-Hyg plasmids (Takara Bio, 632182) containing the target gene coding sequence were used to produce Lentivirus in Lenti-X 293T cells. The empty vector was used as a control. Cancer cells were transduced overnight and fresh medium was replaced the next day. Polyclonal cells were selected with hygromycin at 100 μg/ml for 4T1 and 500 μg/ml for EMT6.5 cells for 72 hours. The confirmation of the genetical modification efficiency was done (Extended Data Figure 6). For fluorescence-activated cell sorting (FACS)-based isolation of cells *in vivo*, 4T1 and EMT6.5 cells were transduced with the pLKO.3 Thy1.1 (CD90.1) vector (Addgene plasmid 1479). CD90.1-positive cells were then isolated using flow cytometry.

#### Animal Studies

The mice were housed in a conventional facility under a 12-hour light/12-hour dark cycle, with ad libitum access to food and water. Temperature was monitored daily and maintained at 22 ± 2 °C, while humidity was also checked daily and kept within the range of 45–70%. Randomization was performed prior to the injection of cancer cells, and all analyses were carried out in a blinded manner. Sample size was determined through power calculations (B = 0.8, P < 0.05) based on preliminary data and adhered to the principles of the 3Rs (Replacement, Reduction, Refinement).

Female BALB/c mice (6–8 weeks old, Envigo) were used for all experiments. For the 4T1 and EMT6.5 metastasis intravenous injection model, shRNA or overexpression cell lines were injected at 1 × 10^5^ cells in 100 µL PBS per mouse, ensuring comparability across pooled experiments. Mice were sacrificed 13 days post-injection for both pooled and validation studies using intraperitoneal injection of 10 µL/g of a ketamine (100 mg/kg) and xylazine (10 mg/kg) mixture for organ dissociation experiments or intraperitoneal injection of approximately 50 µL of a 60 mg/mL Dolethal (pentobarbital sodium) solution (Vetoquinol). Lung dissociation procedures were performed as previously described^15^. For the mammary fat pad (m.f.p.) metastasis model, mice were injected with 50 000 4T1 cells in 50 µL PBS into the m.f.p. region and sacrificed 25 days post-injection. Euthanasia method is intraperitoneal injection of 50 µL of a 60 mg/mL Dolethal solution for metastasis isolation or immunohistochemistry (IHC) staining. Tumor volumes were measured using manual calipers and calculated as 0.5 × length × width × width, with tumors weighed at the end of each experiment. Humane endpoints, monitored using a predefined score sheet, included tumor size exceeding 1.8 cm^3^, impaired ambulation, labored respiration, surgical infection, or weight loss greater than 10% of initial body weight. For all experiments, the maximum permitted tumor size was 1.8 cm^3^, as approved by the ethics committee. This limit was not exceeded in the majority of animals. In a subset of animals, tumor size marginally exceeded this predefined limit at the experimental endpoint on the day of scheduled sacrifice. Animals were closely monitored, showed no signs of distress, and were euthanized immediately upon reaching the endpoint. No animals were maintained beyond the approved humane endpoint.

#### High fat diet mouse model

For the high-fat diet (HFD) experiments, 4-week-old female BALB/c mice were randomly assigned to two groups: a control diet (CD, E15742-33, ssniff Spezialdiäten) or a long-chain HFD (S8655-E220, ssniff Spezialdiäten). The macronutrient composition of the diets was 13% fat (from lard), 27% protein, and 60% carbohydrates for the CD, and 60% fat, 20% protein, and 20% carbohydrates for the HFD. Mice were maintained on their respective diets for 16 weeks prior to experimentation. Body weight was monitored every two weeks, and mice were inspected weekly to assess their welfare.

#### UPTIDER samples

Human tissue samples, including lung metastases and healthy tissues, were obtained through the ethically approved UPTIDER program (KU/UZ Leuven Program for Postmortem Tissue Donation to Enhance Research; Leuven, Belgium, NCT04531696, S70446). Patients with metastatic breast cancer who provided informed consent to participate in UPTIDER were included in this study. Detailed clinicopathological information for each patient in the UPTIDER dataset is presented in Supplementary Table 1.

#### Reagents and Free Fatty Acid (FFA) Preparation

Sodium palmitate, stearic acid and oleic acid were purchased from Sigma-Aldrich. FFAs were supplemented to the culture media at varying concentrations to reach a final concentration of 130 μM, in addition to the FBS. FFA stock solutions were prepared by conjugating the free fatty acids to bovine serum albumin (BSA), as previously described^15, 54^, ensuring a final FFA:BSA ratio of at least 3:1. Control conditions without additional FFAs were prepared using the same 10% w/w BSA stock with ethanol added to match the volume used in FFA stocks. The final concentration of ethanol in the culture media was 0.125% (v/v) in all conditions regardless of whether fatty acids were added or not.

#### Cloning

The cDNA of *Kat2a* was amplified from the reverse-transcribed cDNA of 4T1 cells using gene-specific primers designed with 20–30 bp homology arms matching the pLVX-Hygromycin vector. The PCR product was purified using a GeneJET Gel Extraction Kit – (Thermo Fisher Scientific) and assembled into the linearized pLVX-Hygromycin vector using the Gibson Assembly Master Mix (New England Biolabs) according to the manufacturer’s protocol. The assembled reaction mixture was transformed into chemically competent E. coli cells DH5α(NEB), and positive clones were screened by colony PCR and confirmed by Sanger sequencing. For *Zdhhc20* and *Tm4sf1* WT and Mutant) overexpression vectors, codon optimized gene blocks were synthesized by Integrated DNA Technologies (IDT) with flanking sequences designed to overlap with the linearized pLVX vector for Gibson Assembly.

#### Size exclusion analysis to estimate correct folding of TM4SF1 constructs

cDNAs, encoding wildtype and mutant (C79A/C88A) mouse TM4SF1 (Uniprot accession code Q64302), flanked by SapI sites, were synthesized (Twist Biosciences) and ligated into pcDXC3MS (Addgene #49030), using FX cloning^55^. Two constructs were generated: C-terminally tagged wild type and mutant murine TM4SF1. Plasmids were verified by Sanger sequencing and used for transient transfection of Expi293 cells (Thermo Fisher Scientific).

A suspension culture of Expi293 cells at a density of 2 mio cells/ ml was transfected using linear MW 25000 polyethyleneimine (Polysciences) and plasmid DNA mixed in a 3:1 ratio (wt/wt) in Opti-MEM (Gibco) medium and harvested after three days in aliquots of 5 ml. After harvesting, pellets were frozen using liquid N2 and stored at -80°C. After thawing all subsequent steps were performed at 4°C or on ice. To compare the solubilization and monodispersity as an indicator for protein folding between wild type and mutant TM4SF1, a detergent screen was performed using (final concentrations): 2% glycodiosgenin (GDN, Molecular Dimensions), 2% Dodecyl-β-D-Maltopyranoside (Glycon) with 0.4% cholesterol hemisuccinate (Sigma) (DDM/CHS) and 2% lauryl maltose neopentyl glycol (Molecular Dimensions) with 0.2% cholesterol hemisuccinate (LMNG/CHS), respectively. In brief, proteins from a 5 ml culture were extracted in a buffer containing 50 mM Hepes pH 7.5, 250 mM NaCl, 10% glycerol, protease inhibitors (cOmplete, Roche) and the indicated detergent/lipid mixture for 1 hour on a rotary device. Cell debris and insoluble matter was removed by centrifugation at 15,000g for 30 minutes and the cleared supernatant was applied to streptavidin resin (Thermo Scientific) for 1.5 hours on a rotary device. Unspecific proteins bound to the resin were removed with wash buffer (30 mM Hepes pH 7.5, 150 mM NaCl, 6% glycerol containing 0.0063% GDN, 0.03% DDM/0.006% CHS or 0.003% LMNG/0.0003% CHS, respectively). TM4SF1 proteins were eluted with the same buffers but containing 10 mM biotin. Eluted proteins were applied to SDS-PAGE for Coomassie-staining and fluorescent size exclusion chromatography (FSEC) on an Agilent 1260 Infinity II HPLC using a Superdex 200 Increase 5/150 column (Cytiva) to address monodispersity in the respective detergents and to compare wild type and mutant TM4SF1. The column was equilibrated with 10 mM Hepes pH 7.5, 150 mM NaCl and the corresponding detergents before proteins were injected.

#### Bioinformatic analysis

Gene Set Variation Analysis (GSVA) was performed to evaluate the activity of the STAT3 or DDR1 signaling pathway and its correlation with *KAT2A* expression in RNA-seq data from metastatic breast cancer samples (cBioPortal). The STAT3 gene signature was obtained from the Molecular Signatures Database (MSigDB). The DDR1 signature was defined based on the RNA-seq dataset from E0771 *Ddr1* knockdown experiments^56^. The top 100 significantly downregulated genes in the *Ddr1* knockdown condition were selected to construct the DDR1 signature. RNA-seq data were normalized using the log2 transformation method. GSVA scores were calculated with parameter set as “kcdf = Gaussian” using the GSVA(1.52.3) R package^57^. Pearson correlation analysis was conducted to investigate the relationship between the GSVA-derived STAT3 pathway scores and normalized *KAT2A* expression levels. The correlation coefficient (r) and p-value were calculated.

Gene Set Enrichment Analysis (GSEA) was performed to evaluate the enrichment of the STAT3 and DDR1 signatures in microarray data(GSE14018) from metastatic breast cancer patients, comparing lung and brain metastases. Differential expression analysis between lung and brain metastases was performed using the limma(3.54.2) R package^58^. Genes were ranked by their log2 fold change, and the resulting ranked gene list was used as input for GSEA. For the breast cancer patients with lung metastasis(GSE193103), patients were stratified into high and low *ZDHHC20* expression groups based on the median expression level. Differential expression between high and low *ZDHHC20* expression groups was analyzed using the limma R package^58^. GSEA was conducted using the clusterProfiler(4.6.2) R package with the GSEA function^59^. Enrichment scores (ES), normalized enrichment scores (NES), and false discovery rate (FDR)-adjusted p-values were calculated to assess the significance of enrichment for both the STAT3 and DDR1 signatures. All analyses were performed in R (version 4.2.3), and results were visualized using the ggplot2(3.5.1) package.

#### Multiplex immunohistochemistry staining

Human and mouse tissues were fixed in 10% formalin for 24 hours, paraffin-embedded, and sectioned at 4 µm. The formalin-fixed paraffin-embedded (FFPE) sections were deparaffinized in xylenes, rehydrated in ethanol, and subjected to heat-mediated antigen retrieval in BOND Epitope Retrieval Solution 2 (AR9640) for 10 minutes in a pressure cooker. After cooling, the slides were blocked with blocking buffer (10% goat serum, Invitrogen, 0.5% BSA in TBS Buffer) for 30 minutes at room temperature and incubated with the primary antibody for 30 minutes at room temperature, followed by three washes with TBS/T for 3 minutes each. Primary antibodies were diluted in TNB (Tris-NaCl Blocking buffer): 100 mM of Tris-HCl pH 7.5, 150 mM NaCl, 0.5% w/v of blocking reagent (Akoya, FP1020). The slides were then incubated with secondary antibody-HRP (Dako rabbit or Opal kit for mouse HRP) for 10 minutes at room temperature and washed again with TBS/T three times for 3 minutes each. Afterward, the slides were stained with Opal fluorophore working solution (Akoya Biosciences) for 10 minutes. Heat-mediated antibody stripping was performed to remove primary and secondary antibodies, allowing for additional rounds of labeling with other primary antibodies. Slides were counterstained with 1 ng/mL DAPI (Spectral DAPI Akoya) for 10 minutes at room temperature and mounted with coverslips using Immu-Mount mounting medium (Thermo Fisher Scientific, 9990402). The antibody specificity and dilution were verified before the multiplex assay. Slides were imaged using the AKOYA Phenoimager and analyzed using Qupath.

Quantification of p-STAT3 and KAT2A in mouse tissue was performed by calculating the ratio of KAT2A-positive cells to EPCAM-positive cells within metastases. Five randomly selected metastases from a single mouse were analyzed, and the results were averaged to represent one replicate. For human tissue, quantification of p-STAT3, KAT2A, and ZDHHC20 was based on the fluorescent intensity per unit area. Measurements were taken from five randomly selected regions within each metastatic (PanCK-positive) or adjacent (PanCK-negative) tissue. For each patient, five random metastases were selected, and the averaged values were used as one replicate.

#### Hematoxylin and eosin staining

Hematoxylin and eosin (H&E) staining of pulmonary metastases was performed as previously described^19^. Briefly, dissected lung samples were gently infused via the trachea with 10% formalin, fixed overnight, and immersed in 70% ethanol for 24 hours. Then, 5-μm thick sections obtained from the resulting paraffin blocks were stained with haematoxylin and eosin. Scanned slides were analyzed for metastatic area using Qupath software. Metastatic burden was determined based on the metastatic area or metastatic index (metastatic area divided by primary tumor weight).

#### Immunofluorescence

After 5 days of culture 3D spheroids or 2D cells were transferred to fibronectin-coated plates and allowed to loosely attach to facilitate the following steps. The spheroids were fixed with 4% paraformaldehyde in PBS for 30 min and permeabilized with 0.5% Triton X100 in PBS for 30 min. The 2D cells were starved with serum-free media for 12 hours and then fixed with 4% paraformaldehyde in PBS for 10min and no permeabilization is needed for membrane marker staining. The spheroids were then incubated with phosphor-STAT3 (PY705, Cell Signaling 9198; 1:200 dilution) or STAT3(Proteintech 10253-2-AP; 1:200 dilution)) antibodies overnight at 4°C, washed 3 times with PBS, incubated for 1h with the appropriate Alexa Fluor 555-conjugated secondary antibody (Life Technologies, A31272) and then washed again 3 times in PBS.c Nuclei were stained with a solution of 6 μM of 4’,6-diamidino-2-phenylindole (DAPI; Sigma Aldrich, D9542) in PBS for 15 min. Coverslips were mounted in Fluorescence Mounting Medium (Dako, S3023). The samples were visualized on a SP8X or SPE inverted confocal microscope (Leica Microsystems) equipped with a 405 nm and a white light laser (SP8x) or a 561 laser (SPE). Images were acquired in form of Z-stacks using the LAS AF acquisition software (Leica Microsystems).

#### Flow cytometry

For quantification of CD90.1 positive cancer cells in the lung metastasis, lung dissociation was performed to have single cell suspensions. Then 30 × 10^6^ cells/mL were preincubated with anti-mouse CD16/CD32 (Fc block; BD Biosciences) before staining for 20 min at 4 °C for flow cytometry analysis. Antibodies against CD45 (BD Biosciences, 550994; 1:250), PDPN (BioLegend, 127409; 1:250), and CD90.1 (BioLegend, 202505; 1:400) were used to identify cancer cells within murine lung single-cell suspensions. Dead cells were excluded using Viability eFluor450 (Thermo Fisher, 65-0863-14; 1:500). Samples were analyzed on the BD FACSymphony A1 using FACSDiva software (BD Biosciences). Metastatic burden was quantified as the proportion of CD90.1-positive cells in the lung by FlowJo software (Supplementary Figure 1).

For quantification of TM4SF1 plasma membrane staining of non-permeabilized cells, 4T1 cells were seeded on 6-well plates overnight. The following day, cells were washed with PBS and incubated in FBS-free medium for 4h at 37 °C. They were then treated with either BSA or 75 μM palmitate for 2 hours at 37 °C, followed by three washes with PBS containing 1% BSA and one wash with PBS. Afterwards, cells were trypsinized and incubated at 4 °C for 10 min with anti-mouse CD16/CD32 (Fc block, BD Biosciences, 553142), and stained with the appropriate antibody on ice for 15 min. Anti-mouse TM4SF1 (R&D systems, AF7514) and sheep IgG isotype control (R&D systems, 5-001-A) with anti-sheep 488 secondary (Abcam, ab150177) were used at a 1:50 dilution. Cells were then washed three times with 2 mL FACS buffer, filtered through a cell strainer, and analyzed on the BD FACSymphony A1. Data were analyzed using FlowJo software (Supplementary Figure 1).

#### In vitro binding assay

An antibody-based pull-down assay was performed to examine the interaction between recombinant DDR1 and TM4SF1. Protein A/G magnetic beads(Invitrogen, 10008D) were pre-incubated with an anti-DDR1 antibody(Proteintech, 10536-1-AP), followed by binding of recombinant DDR1 protein(Bio-Connect, HY-P77639) to the antibody-coated beads. The DDR1–bead complexes were then incubated overnight at 4 °C with lysates from 4T1 cells containing TM4SF1(wild-type or C79-88A mutant). After extensive washing, bound proteins were eluted in SDS loading buffer and analyzed by SDS-PAGE and Western blotting.

#### Western blotting

Original western blot membranes are available as Supplementary Information. Spheroids were collected and then lysed in RIPA lysis and extraction buffer supplemented with proteinase and phosphatase with the help of a tissue lyser. For cell fractionation to enrich nuclear fraction, it was performed as previously described^15^. Protein amount was measured using a pierce BCA protein assay kit. 20-30 µg of protein were loaded on a NuPAGE 4–12% denaturing BisTris gel and transferred to a nitrocellulose membrane. Membranes were incubated overnight at 4°C with either KAT2A (Cell Signaling 3305; 1:2000 dilution), DHHC20 (Sigma HPA014702; 1:1000 dilution), phosphor-STAT3 (PY705, Cell Signaling 9145; 1:1000 dilution), STAT3 (Cell Signaling 9139, Proteintech 10253-2-AP; 1:1000 dilution), Flag(Proteintech, 20543-1-AP), NF-κB p65-Acetyl (Abcam, ab19870), NF-κB p65(Cell Signaling, 8242S), Histone H3(Abcam, ab1791), Vinculin(Cell Signaling, 13901S) and β-ACTIN (Sigma A5441; 1:10000 dilution) primary antibodies. The day after the membranes were incubated with either mouse (Cell Signaling Technology, 7076; 1:5000 dilution) or rabbit (Cell Signaling Technology, 7076; 1:5000 dilution) secondary antibodies, and bound antibodies were visualized using SuperSignal West Pico PLUS Chemiluminescent Substrate (Invitrogen, 34580) or SuperSignal West Femto Maximum Sensitivity Substrate (Invitrogen, 34095). The membranes were stripped with Western Blot Stripping Buffer (Thermo Fisher, 46430) for another round of antibody incubation and ECL visualization until all proteins were detected.

#### RNA isolation and RT-PCR

Total RNA was isolated with TRI reagent. 500-1000 ng of RNA was reverse transcribed into cDNA using a qScript cDNA Synthesis Kit. The relative levels of transcripts compared to the control housekeeping gene (Ribosomal Protein L19, *RPL19*) were determined by qPCR using PerfeCTa SYBR Green SuperMix, Low ROX and specific primers on a 7500 Fast Real Time PCR System. Amplification was performed at 95 °C for 10 min, followed by 40 cycles of 15 s at 95 °C and 1 min at 60 °C.

#### ABE assay

In the IP-ABE assay, immunoprecipitation is used to isolate Flag-TM4SF1 proteins, and this procedure is followed by the ABE chemical reaction^60^. The multistep IP-ABE assay was performed as follows: After 3 days of 3D colony culture, cells were treated with lysis buffer (50% RIPA buffer with 1% ASB14 and 0.1% Triton-100x + 50% Lysis buffer, ph=7.4 (50 mM Tris-HCl pH 7.5, 150 mM NaCl, 1 mM MgCl_2_, 1% NP-40, 10% glycerol with protease inhibitor and phosphatase inhibitor)) containing 50 mM N-ethylmaleimide (NEM) to have the protein lysate. For immunoprecipitation, 3 µL of Flag antibody (Proteintech, 20543-1-AP) was added to the samples per 500 µg of protein and incubated overnight at 4 °C. The supernatants were mixed with 35 μL of a 50% slurry of magnetic beads with incubation for 4 h on the rotation wheel at 4 °C. Next, immunoprecipitants were treated with 1 M hydroxylamine (HAM) in lysis buffer (ph=7.2) for 1 h at room temperature, to selectively cleave the cysteine residues that were palmitoylated. The HAM-treated beads were incubated with 5 μM biotin-BMCC (ph=6.2) for 1 h at 4°C. Immunoprecipitated protein samples were eluted from the beads by treatment with 2X SDS sample buffer containing no reducing agents. Protein samples were loaded onto SDS-PAGE for Western blot analysis. Palmitoylation levels were detected using streptavidin-HRP, while total protein levels were assessed using a Flag antibody.

#### Chromatin immunoprecipitation (ChIP) qPCR

4T1 and EMT6.5 cells were seeded in 10cm 0.5% agar dishes for 3D culture in RPMI supplemented with 10% FBS and 1% penicillin/streptomycin with or without 75 μM palmitate supplementation. The following day, ChIP of STAT3 was performed according to a standard ChIP protocol^61^ using 25μg of chromatin and 3μg of either anti-STAT3 ChIP antibody (Cell Signaling 9139 or Proteintech 10253-2-AP) or anti-rabbit IgG (Cell Signaling 2729).Chromatin was immunoprecipitated overnight at 4°C and STAT3 binding sites assayed via RT-qPCR using 2μl of either input, IgG-IP, or STAT3-IP DNA per reaction in triplicate wells per primer pair. Primers targeting a gene desert region, a known STAT3 site in the *Fascin* promoter^62^, or a predicted STAT3 binding site (JASPAR) in the *KAT2A* promoter were used to confirm binding (Supplementary Table 2).

#### Statistics and reproducibility

All statistical analyses were conducted using GraphPad Prism 10 (GraphPad Software) with at least three biological replicates (n ≥ 3). Specific statistical tests and post hoc analyses are described in the figure legends, and non-significant comparisons are not shown. Data are reported as mean ± s.d. Statistical outliers were identified using Grubb’s test. Animals were excluded if they died or had to be killed according to ethical protocols. In Extended Data Figure 5d tumor sizes were excluded that could not accurately be measured by caliper (see source data). For in vitro experiments, no data were excluded. Sample sizes for *in vitro* experiments were determined empirically, while sample sizes for *in vivo* studies were calculated based on power analysis (β = 0.8, P < 0.05) using preliminary data. For in vivo experiments, mice were randomized prior to diet administration or injection with different cell lines. Animals were assigned unique identification numbers before data collection to enable blinded analysis. For in vitro studies, samples were randomized prior to data acquisition whenever possible. Data distribution was assumed to be normal, but this was not formally tested. Individual data points are shown where applicable to illustrate data distribution.

#### Lead Contact

Further requests for resources should be directed to the lead contact, Sarah-Maria Fendt (sarah-maria.fendt@kuleuven.be).

#### Materials Availability

This study did not generate new unique reagents, except of genetically manipulated cell lines based on commercially available constructs. Reagents generated in this study will be made available on request through the lead author or the collaboration partner that generated the resource, but we may require a payment and/or a completed Materials Transfer Agreement if there is potential for commercial application.

## Extended Data

**Figure:**
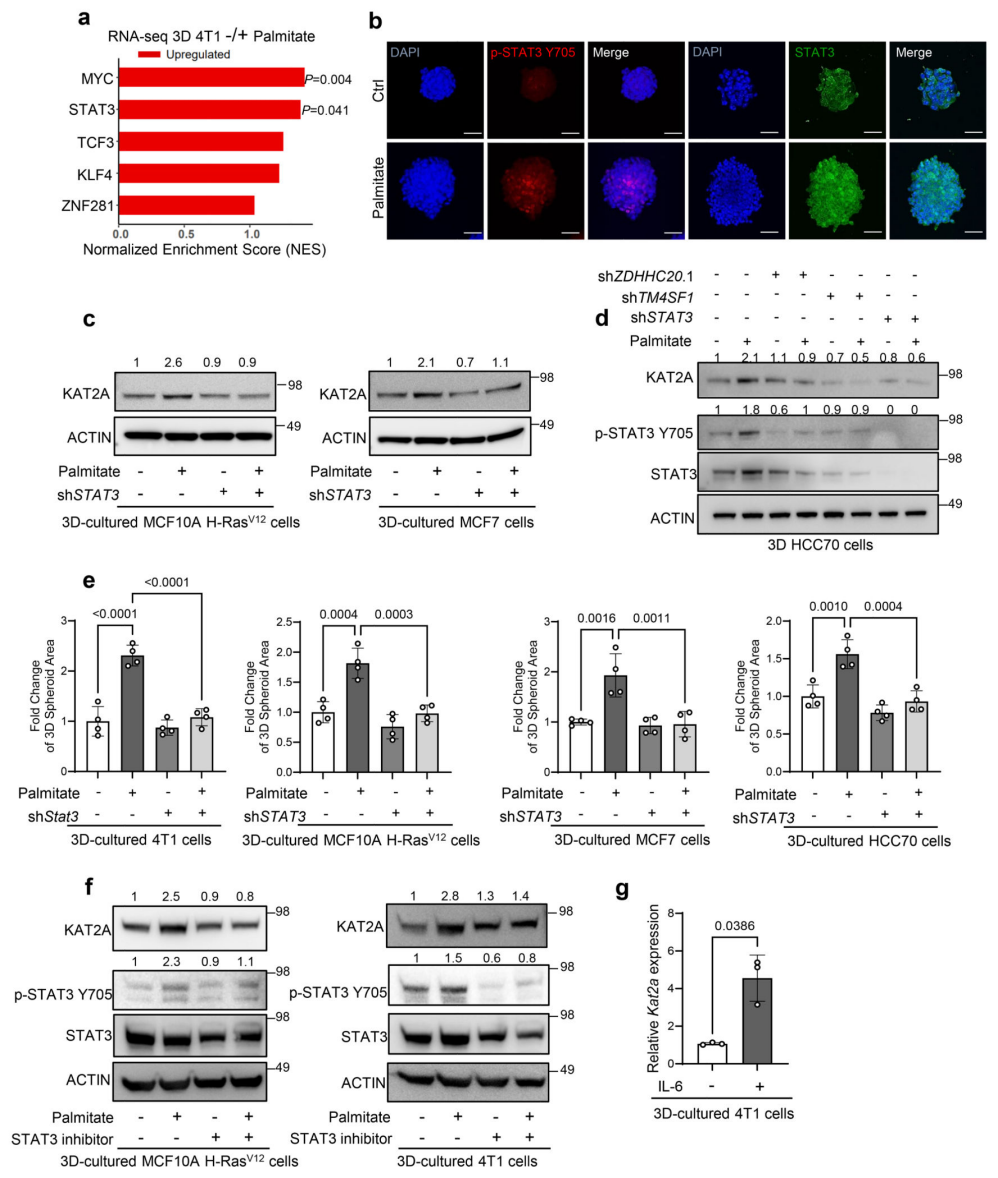


**Figure:**
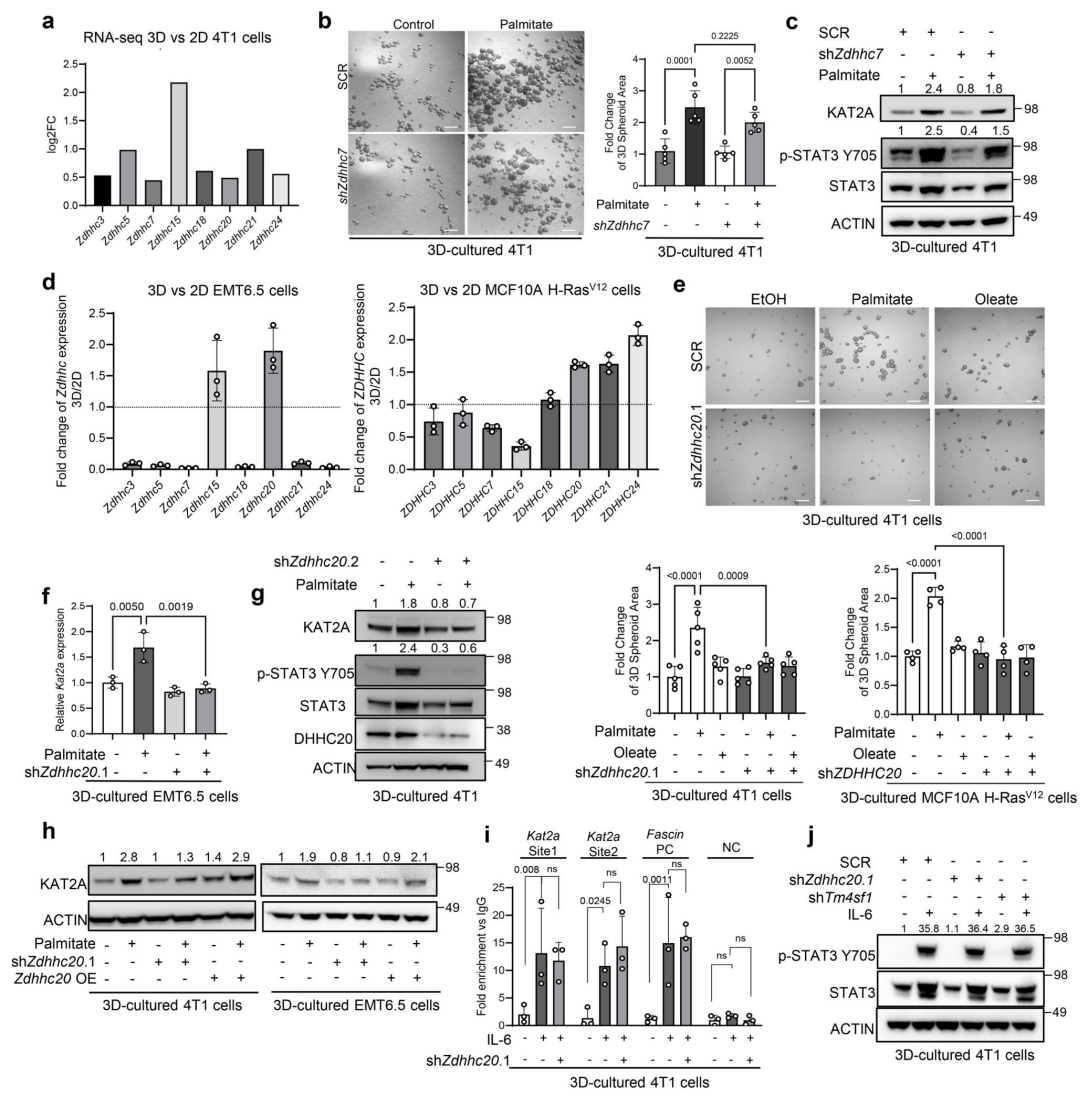


**Figure:**
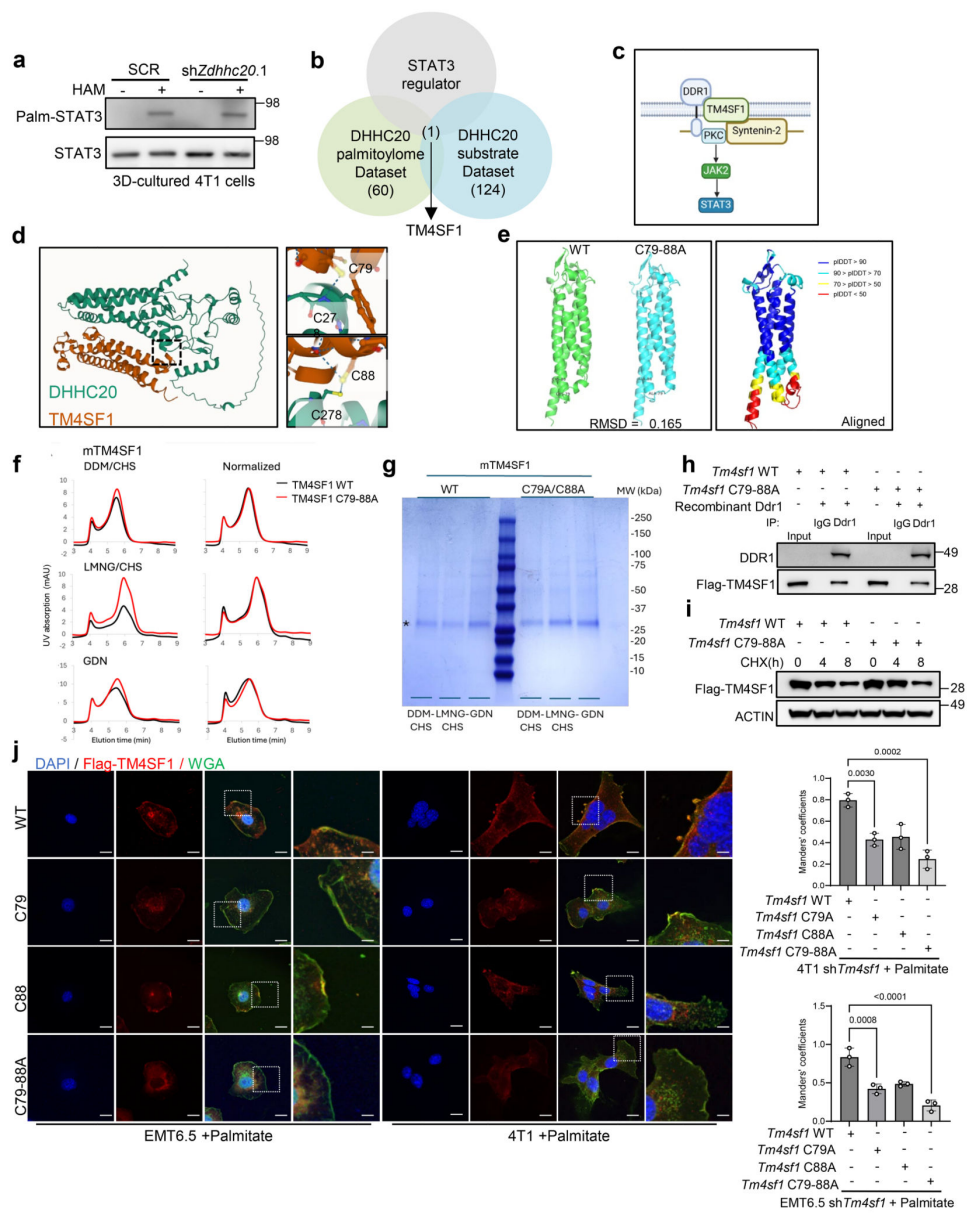


**Figure:**
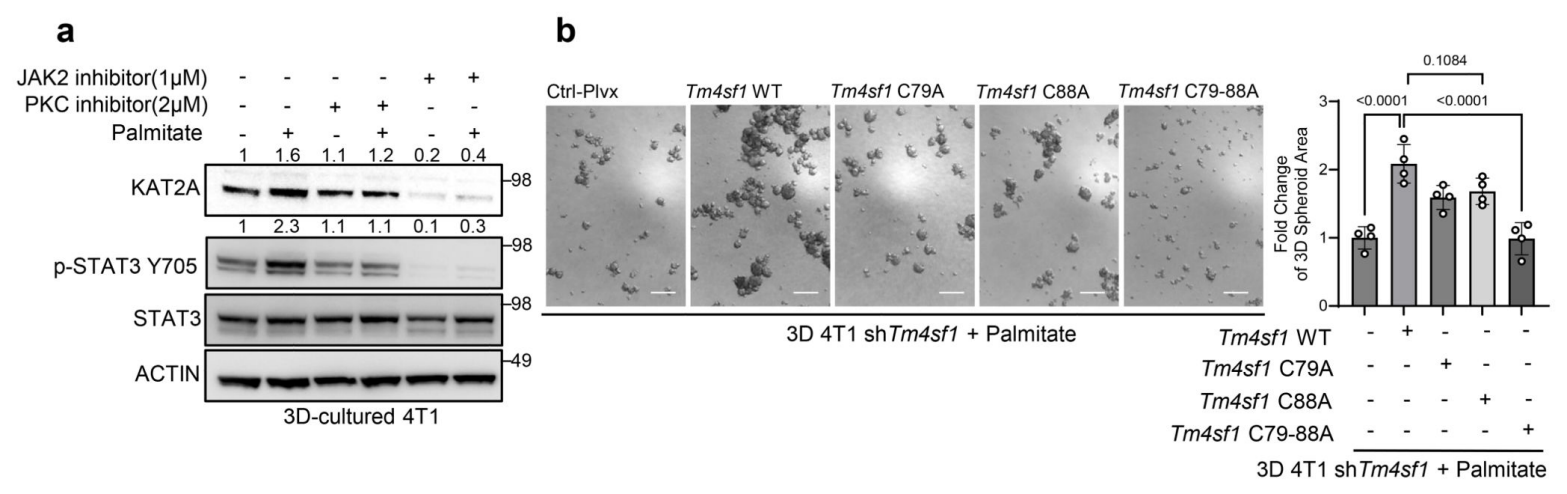


**Figure:**
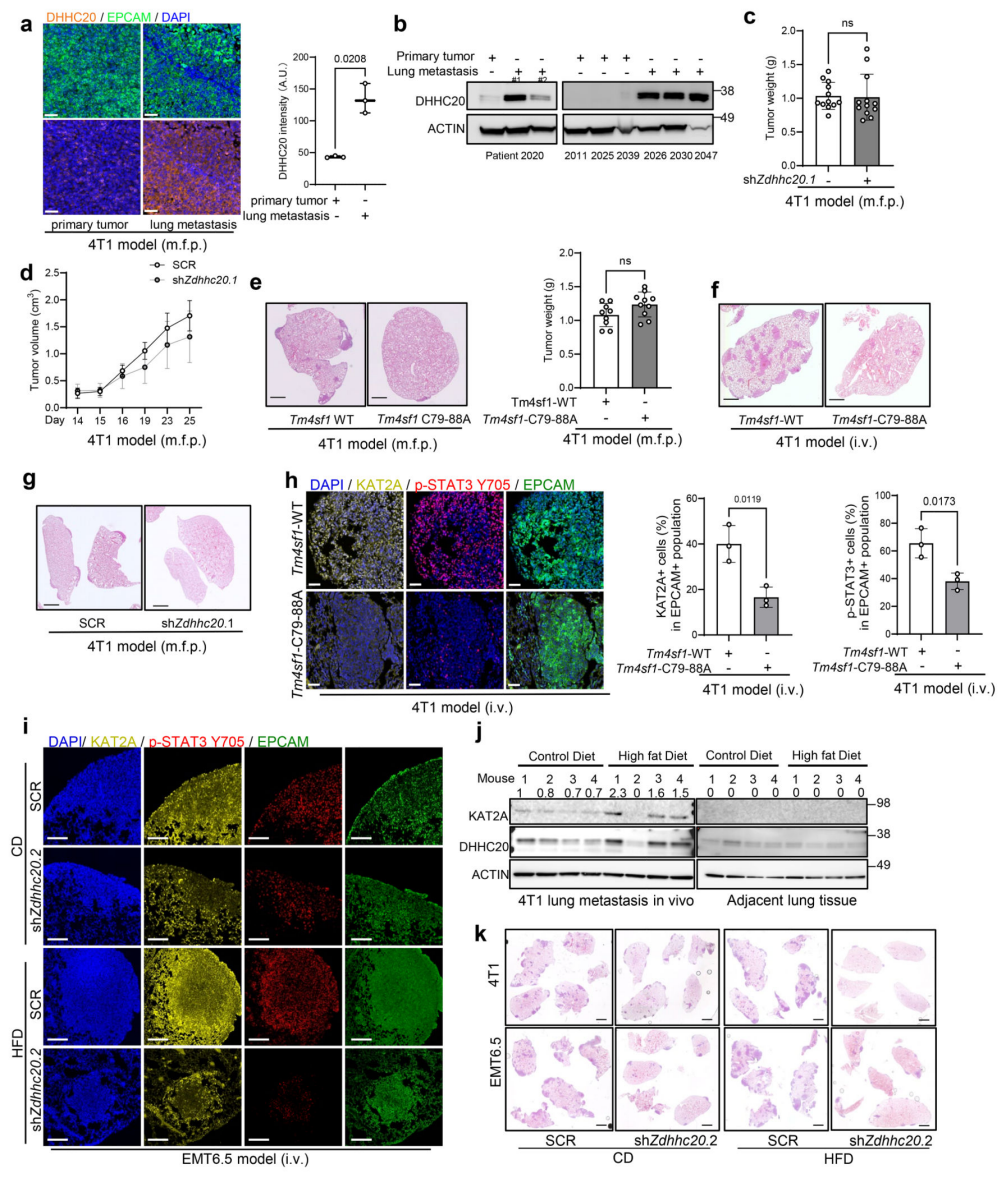


**Figure:**
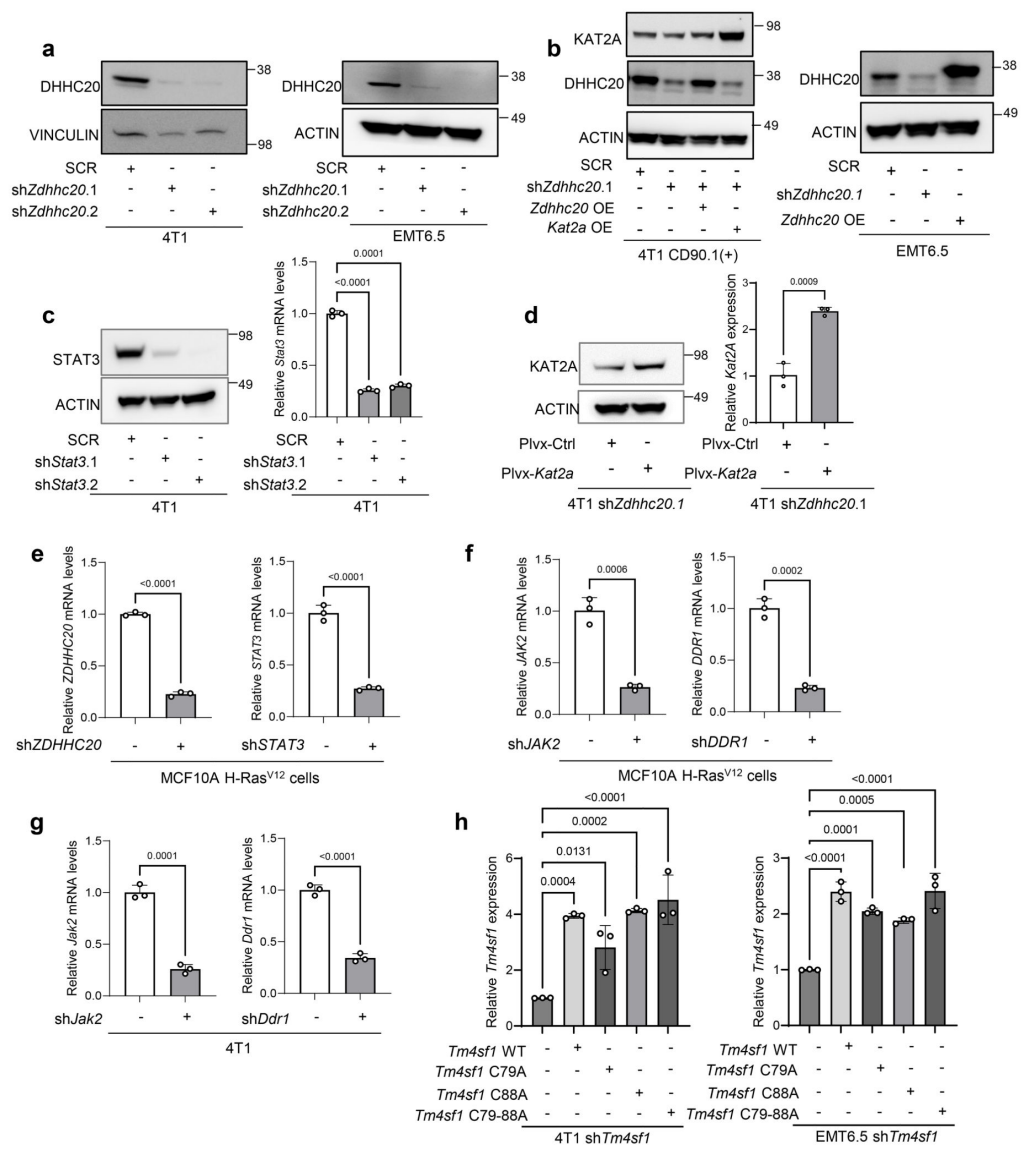


## Supplementary Material

Supplementarty Table 1

Supplementarty Table 2

Supplementary Figure 1

## Data Availability

The publicly available microarray-based patient-metastasis data set GSE14018 can be downloaded from the Gene Expression Omnibus (GEO) https://www.ncbi.nlm.nih.gov/geo/query/acc.cgi?acc=GSE14018. The RNA-seq breast cancer patients with metastasis dataset GSE193103 can be downloaded from the GEO https://www.ncbi.nlm.nih.gov/geo/query/acc.cgi?acc=GSE193103. The 4T1 3D palmitate treatment dataset GSE197134 can be downloaded from the GEO https://www.ncbi.nlm.nih.gov/geo/query/acc.cgi?acc=GSE197134. The metastatic breast cancer patient database is downloaded from http://www.cbioportal.org/study/summary?id=brca_mbcproject_2022. All other data supporting the findings of this study are available within the Article and the Supplementary Information, and from the corresponding author on reasonable request.

## Code Availability

No original software and/or algorithms were developed in the present study; however, code used for data analysis can be provided upon request. Any additional information required to reanalyze the data reported in this paper is available from the corresponding author upon request.
