# Supplementary material for: Palmitoylation-mediated regulation of KAT2A promotes lung metastasis in breast cancer
Source: Nat Cell Biol. Author manuscript; Available in PMC 2026 Apr 4. (PMC7618962; doi:10.1038/s41556-026-01913-z)

SUPPLEMENTARY Figure 1: a Gating strategy for TM4SF1-positive 4T1 cells. **b** Gating strategy for CD90.1-positive 4T1 cells from lung pf female BALB/c mice.

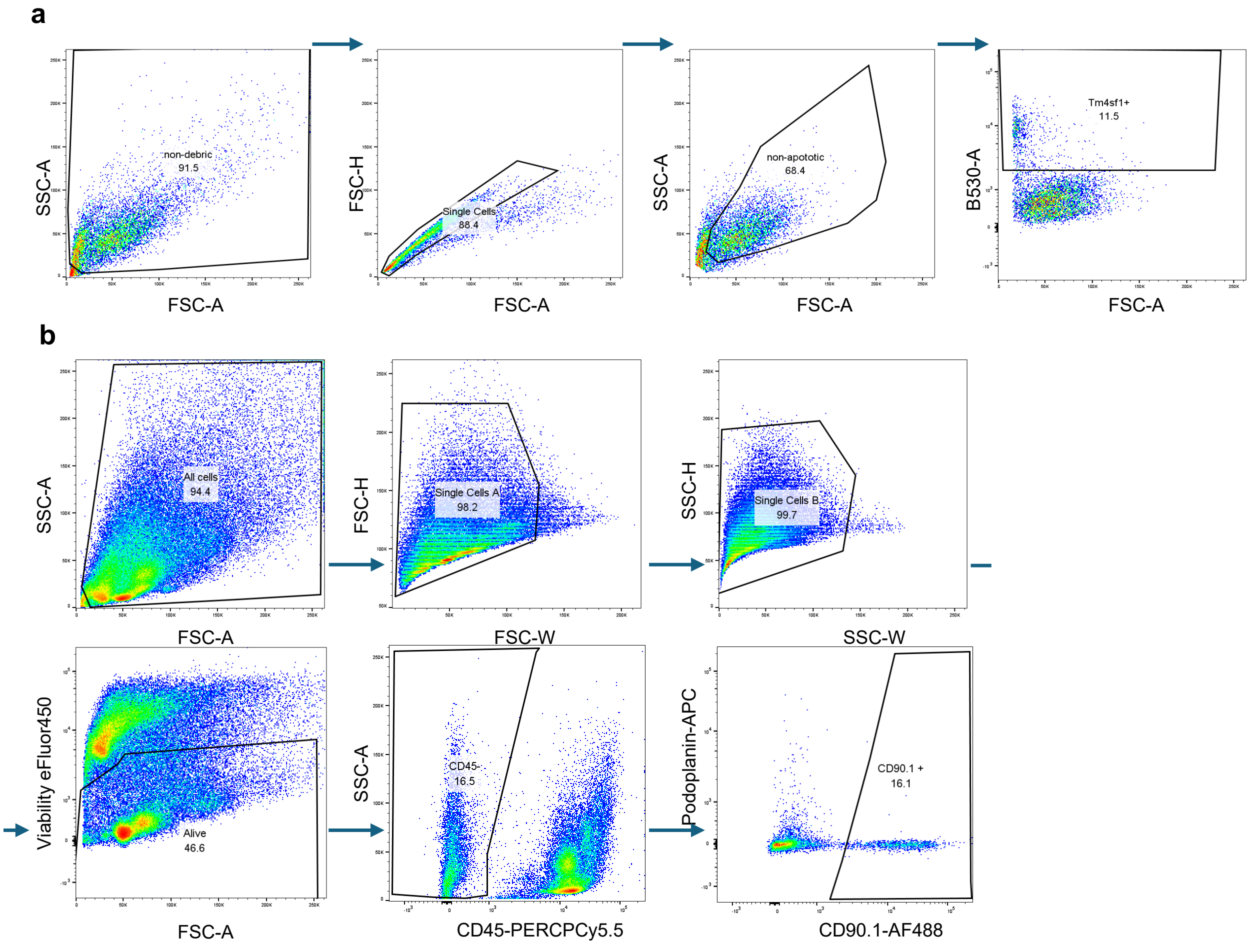

Supplement: Supplementary Figure 1 [file EMS212993-supplement-Supplementary_Figure_1.pdf]
